# Ultraconserved element uc.372 drives hepatic lipid accumulation by suppressing miR-195/miR4668 maturation

**DOI:** 10.1038/s41467-018-03072-8

**Published:** 2018-02-09

**Authors:** Jun Guo, Weiwei Fang, Libo Sun, Yonggang Lu, Lin Dou, Xiuqing Huang, Weiqing Tang, Liqing Yu, Jian Li

**Affiliations:** 1 The MOH Key Laboratory of Geriatrics, Beijing Hospital, National Center of Gerontology, Beijing, 100730 China; 2 Graduate School of Peking Union Medical College and Chinese Academy of Medical Sciences, Beijing, 100730 China; 3 Department of Hepatobiliay Surgery and You-An Liver Transplantation Center, Beijing You-An Hospital, Capital Medical University, Beijing, 100069 China; 4 Center for Molecular and Translational Medicine, Institute for Biomedical Sciences, Georgia State University, Atlanta, GA 30303 USA

## Abstract

Ultraconserved (uc) RNAs, a class of long non-coding RNAs (lncRNAs), are conserved across humans, mice, and rats, but the physiological significance and pathological role of ucRNAs is largely unknown. Here we show that uc.372 is upregulated in the livers of db/db mice, HFD-fed mice, and NAFLD patients. Gain-of-function and loss-of-function studies indicate that uc.372 drives hepatic lipid accumulation in mice by promoting lipogenesis. We further demonstrate that uc.372 binds to pri-miR-195/pri-miR-4668 and suppresses maturation of miR-195/miR-4668 to regulate expression of genes related to lipid synthesis and uptake, including *ACC*, *FAS*, *SCD1*, and *CD36*. Finally, we identify that uc.372 is located downstream of the insulinoma-associated 2 (*INSM2*) gene that is transcriptionally activated by upstream transcription factor 1 (*USF1*). Our findings reveal a novel mechanism by which uc.372 drives hepatic steatosis through inhibition of miR-195/miR-4668 maturation to relieve miR-195/miR-4668-mediated suppression of functional target gene expression.

## Abstract (web summary)

Ultraconserved RNAs are a class of long non-coding RNAs whose functions are yet to be identified. Here Guo and colleagues show that an ultraconserved RNA uc.372 promotes lipogenesis and lipid accumulation within the hepatocytes by suppressing the maturation of miR-195/miR-4668 that inhibits lipogenic gene expression.

## Introduction

As the most prevalent chronic liver disease, the pathogenesis of non-alcoholic fatty liver disease (NAFLD) is complicated and multi-faceted. According to the double-hit theory, the first hit of NAFLD is the accumulation of abnormal triglycerides in hepatocytes, thereby resulting in the second hit in which inflammatory mediators further lead to liver injury, inflammation, and fibrosis^1,2^. To improve our understanding of NAFLD pathogenesis, ongoing efforts has been mainly focused on protein-coding genes^3–5^. However, the majority of the human genome produces functional non-coding RNA^6^. Increasing evidence suggests important biological, developmental, and pathological roles for non-coding RNA through mechanisms, including cis regulation at enhancers, chromatin reprogramming, and mRNA processing at the posttranscriptional level^7,8^.

Although long non-coding RNAs (lncRNAs) were previously considered to be transcriptional “noise,” accumulating evidence has identified a biological function for lncRNAs^9,10^. Recent studies raise the possibility that the appearance of lncRNAs plays an important regulatory role in the pathogenesis of human diseases^11,12^. However, the functional role of lncRNAs remains largely unknown^13^.

LncRNAs, particularly highly conserved ones, are postulated to undergo active regulation and may have a cellular function^14,15^. The ultraconservation of some lncRNAs came to light after a genome-wide survey identified 481 lncRNAs longer than 200 bp in length with 100% identity across the human, mouse, and rat genomes^16^. These highly conserved lncRNAs have been termed ultraconserved elements (UCEs)^16^. Functional analysis indicates that UCEs are distributed in clusters in regions that are predicted to code for transcription factors and developmental genes^17,18^. These ultraconserved regions have been suggested to play critical roles in DNA binding, RNA processing, and transcription^17,19^. However, our understanding of these elements remains limited. Recently, it has been shown that the expression pattern of transcribed ultraconserved RNAs (ucRNAs) is altered in many human tumors, indicating their potential involvement in cancer progression^20–22^. For example, aberrant expression of UCE 73 (uc.73) regulates apoptosis and cellular proliferation in colorectal cancer cells^20^. Several ucRNAs are tightly correlated with clinical prognostic factors in neuroblastoma^23^. Despite these emerging findings, we still lack knowledge about the functional role of ucRNAs in NAFLD progression. In this study, we suggested a novel mechanism by which uc.372 drives hepatic steatosis through inhibition of miR-195/miR-4668 maturation to relieve miR-195/miR-4668-mediated suppression of functional target gene related to lipid synthesis, including acetyl-CoA carboxylase (ACC), fatty acid synthase (FAS), stearoyl-CoA desaturase 1 (SCD1), and genes related to lipid uptake such as *CD36*, leading to hepatic lipid accumulation. We propose that uc.372 inhibitors may represent potential therapeutic agents for NAFLD.

In sum, therefore, we have identified an ucRNA-based miRNA maturation regulatory pathway that promotes lipid accumulation in liver cells, highlighting the role that ucRNAs play in NAFLD progression.

## Results

### Upregulation of uc.372 in the livers of db/db and high-fat diet-fed mice

**Fig. 1:**
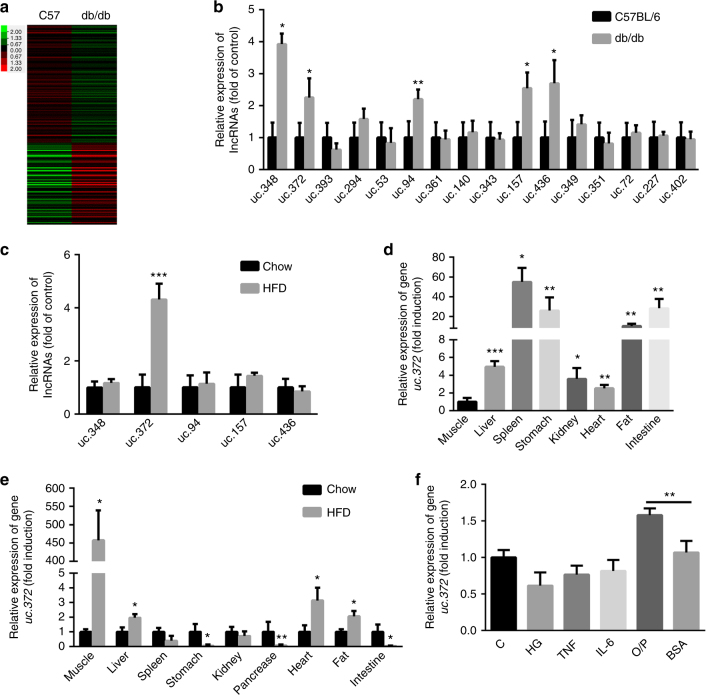
Identification of uc.372 upregulated in the livers of db/db mice and HFD-fed mice. **a** LncRNA-wide expression profiling in the liver of 8-week-old male db/db mice (*n* = 5). **b** Expression levels of ucRNAs in the liver of 8-week-old male db/db mice analyzed by real-time PCR (*n* = 5). **c** Expression levels of ucRNAs in the liver of 10-week-HFD-fed mice analyzed by real-time PCR (*n* = 5). **d** Distribution of uc.372 in various tissues of 6- to 8-week-old C57BL/6J mice (*n* = 3). **e** Distribution of uc.372 in various tissues of 10-week-HFD-fed mice (*n* = 5). **f** Expression level of uc.372 in HepG2 cells treated with 33.3 mM high glucose (HG), 20 nM tumor necrosis factor (TNF), 20 nM IL-6, or 300 μM O/P mixture (*n* = 3). Data are mean ± SEM; *P < 0.05; **P < 0.01; ***P < 0.001 vs. control group. (**b**, **c**, **e** Student’s *t*-test; **d**, **f** analysis of variance (ANOVA))

To identify ucRNAs that are potentially involved in hepatic lipid metabolism, we first searched for ucRNAs that are upregulated in the liver of db/db mice. LncRNA-wide expression profiling identified 16 ucRNAs, representing 3% of all ucRNAs analyzed, that were aberrantly increased by ~6.1- to ~30.4-fold in the liver of db/db mice accompanied by abnormal hepatic lipid accumulation, compared to the livers of wild-type (WT) mice (Fig. 1a, Supplementary Fig. 1a, b). We further assessed changes in these identified ucRNAs by real-time PCR analysis. As shown in Fig. 1b, among the identified 16 ucRNAs, 5 ucRNAs (uc.348, uc.372, uc.94, uc.157, and uc.436) were markedly increased in the liver of db/db mice (*n* = 5). Moreover, we determined levels of 5 ucRNAs in the liver of high-fat diet (HFD)-fed mice and found that the level of uc.372, but not uc.348, uc.94, uc.157, and uc.436, was increased by 4.4 ± 0.4-fold in the liver of these mice accompanied by abnormal hepatic lipid accumulation (Fig. 1c and Supplementary Fig. 1c, d). Next, by analysis of uc.372 distribution in various tissues, we found that uc.372 was widely expressed in mouse organs (Fig. 1d). We hypothesized that if lncRNA controls lipid metabolism, its expression pattern might be affected by changes in metabolic state. In mice subjected to a HFD, uc.372 expression was abundantly increased in metabolism-related organs, including muscle, liver, heart, and fat (Fig. 1e), suggesting that uc.372 is modulated by the metabolic milieu in vivo, thereby acting as a potential metabolic regulator in mice.

To determine whether uc.372 is regulated by the metabolic milieu in vitro, human HepG2 hepatocytes were stimulated with 33.3 mmol/L glucose for 48 h, 10 nmol/L tumor necrosis factor (TNF)-α for 24 h, 10 nmol/L interleukin (IL)-6, or 0.25 mmol/L palmitate for 24 h. As shown in Fig. 1f, palmitate, but not glucose, IL-6 or TNF-α, upregulated uc.372 expression in HepG2 cells. Collectively, these results imply that uc.372 may be involved in hepatic lipid metabolism.

### uc.372 drives hepatic lipid accumulation in mice

**Fig. 2:**
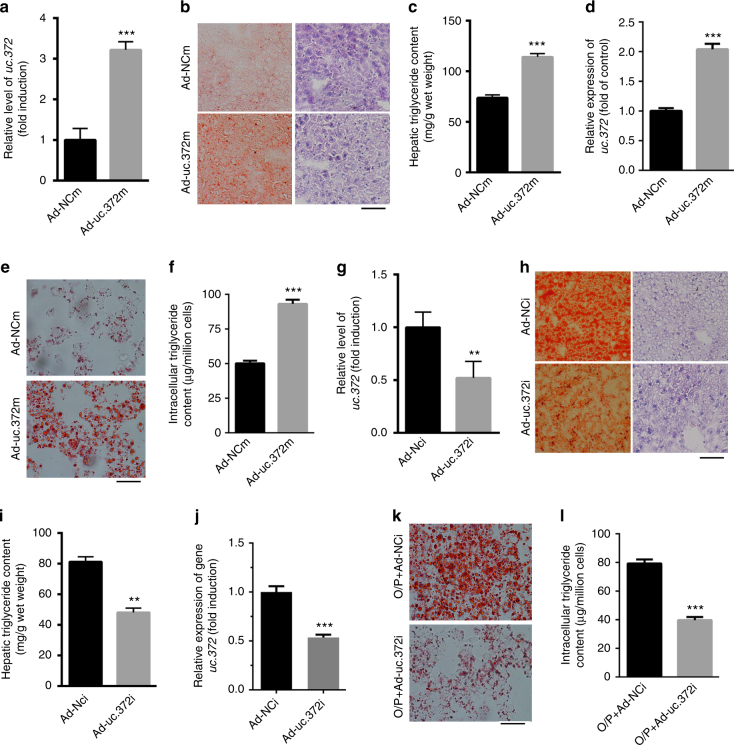
Uc.372 drives hepatic lipid accumulation in mice. **a** Hepatic expression level of uc.372 in 6- to 8-week-old C57BL/6J mice administrated with Ad-uc.372m for 7 days by tail vein injection, as analyzed by real-time PCR (*n* = 5). **b** Representative image from three similar experiments of H&E and Oil Red O staining in liver frozen sections of 6- to 8-week-old C57BL/6J mice after tail vein injection with Ad-uc.372 or Ad-NC for 7 days were shown. Scale bar, 400 μm. **c** Hepatic triglyceride content in 6- to 8-week-old C57BL/6J mice after tail vein injection with Ad-uc.372 or Ad-NC for 7 days (*n* = 5). **d** Expression level of uc.372 in HepG2 cells transfected with Ad-uc.372m or Ad-NC for 48 h (*n* = 3). **e** Representative image from three similar experiments of Oil Red O staining in HepG2 cells transfected with Ad-uc.372m or Ad-NC for 48 h. Scale bar, 40 μm. **f** Intracellular triglyceride content in HepG2 cells transfected with Ad-uc.372m or Ad-NC for 48 h (*n* = 3). **g** Hepatic expression level of uc.372 in 10-week-HFD-fed mice administrated with Ad-uc.372i or Ad-NC for 7 days by tail vein injection (*n* = 5). **h** Representative image from three similar experiments of H&E and Oil Red O staining in liver frozen sections of 10-week-HFD-fed mice after tail vein injection of Ad-uc.372i or Ad-NC for 7 days. Scale bar, 50 μm. **i** Hepatic triglyceride content in 10-week-HFD-fed mice after tail vein injection of Ad-uc.372i or Ad-NC for 7 days (*n* = 5). **j** Expression level of uc.372 in HepG2 cells transfected with Ad-uc.372i or Ad-NC for 48 h (*n* = 3). **k** Representative image from three similar experiments of Oil Red O staining in HepG2 cells transfected with Ad-uc.372i or Ad-NC for 48 h. Scale bar, 40 μm. **l** Intracellular triglyceride content in HepG2 cells transfected with Ad-uc.372i or Ad-NC for 48 h (*n* = 3). Data are mean ± SEM; **P < 0.01; ***P < 0.001 vs. control group (**a**, **c**, **g**, **i**, **j**, **l** Student’s *t*-test)

Next, we hypothesized that hepatic uc.372 upregulation might contribute to aberrant lipid accumulation. To test this idea, uc.372 was overexpressed in the liver of C57BL/6J mice using recombinant adenoviral vectors (Ad-uc.372 m). Seven days after administration of Ad-uc.372m to mice by tail vein injection, uc.372 was overexpressed in the liver of C57BL/6J mice, as indicated by real-time PCR (Fig. 2a). Of note, significantly elevated hepatic triglyceride levels, liver weight, ratio of liver weight to body weight, serum cholesterol, and triglyceride levels (Fig. 2b, c, Supplementary Fig. 2a–c) occurred in Ad-uc.372m-treated C57BL/6J mice. We further investigated whether increased expression of uc.372 in hepatocytes could enhance lipid accumulation. As expected, adenovirus-mediated overexpression of uc.372 in HepG2 cells (Fig. 2d) led to lipid accumulation, as shown by Oil Red O staining and triglyceride content quantification in hepatocytes (Fig. 2e, f). Moreover, we examined the expression of uc.372 in different tissues after tail vein injection with Ad-uc.372m by northern blot. Ad-uc.372m injection led to high expression of uc.372 in the liver, but not in other tissues (Supplementary Fig. 2g). These data suggest that uc.372 overexpression causes severe hepatic lipid accumulation in C57BL/6J mice.

To further assess the role of uc.372 in lipid accumulation, we suppressed uc.372 expression in the liver of HFD-fed mice by recombinant adenoviral vectors expressing an uc.372 inhibitor (Ad-uc.372i) (Fig. 2g). Importantly, we found significantly reduced hepatic triglyceride levels, liver weight, ratio of liver weight to body weight, serum cholesterol, and triglyceride levels in HFD-fed mice injected with Ad-uc.372i (Fig. 2h, i, Supplementary Fig. 2d–f). We next inhibited uc.372 expression by transfection of Ad-uc.372i into HepG2 cells pre-treated with a mixture of oleic acid and palmitic acid (O/P mixture, 2:1, M/M) for 24 h (Fig. 2j). As indicated in Fig. 2k, l, decreased uc.372 expression in hepatocytes attenuated the lipid accumulation induced by the O/P mixture. These results reveal that inhibition of uc.372 has profound lipid-lowering effects in the hepatocytes of HFD-fed mice.

### uc.372 promotes lipid synthesis and uptake in hepatocytes

**Fig. 3:**
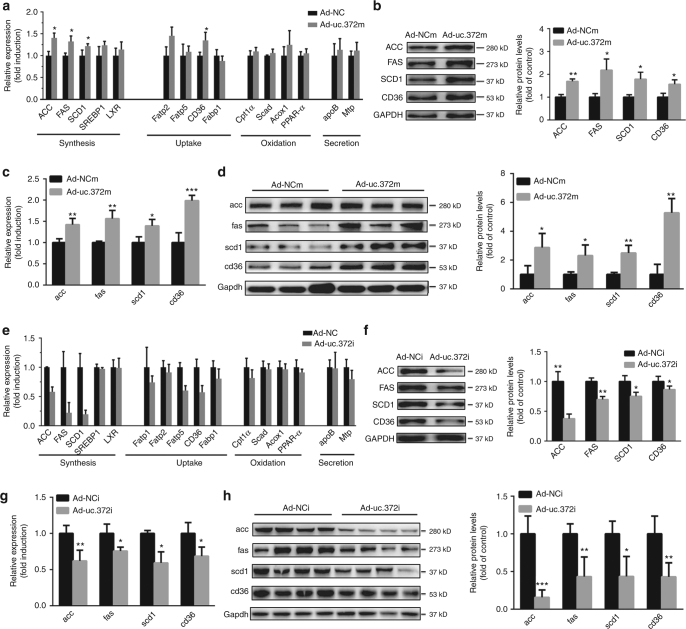
Uc.372 promotes the expression of genes related to lipid synthesis and uptake in hepatocytes. **a** Expression levels of genes related to lipid synthesis (*ACC*, *FAS*, *SCD*, *SREBP1*, and *LXR*), uptake (*Fatp1*, *Fatp2*, *Fatp5*, *Fabp1*, and *CD36*), oxidation (*Cpt1a*, *Scad*, *Acox1*, and *PPAR-a*), and secretion (*apoB* and *Mtp*) in HepG2 cells transfected with Ad-uc.372m or Ad-NCm for 48 h (*n* = 3). **b** Protein levels of ACC, FAS, SCD1, and CD36 in HepG2 cells transfected with Ad-uc.372m or Ad-NCm for 48 h, as analyzed by western blot (representative blots from three similar experiments) (*n* = 3). **c** Expression levels of *acc*, *fas*, *scd1*, and *cd36* in the liver of 6- to 8-week-old C57BL/6J mice injected with Ad-uc.372m or Ad-NCm for 7 days (*n* = 3). **d** Protein levels of acc, fas, scd1, and cd36 in the liver of 6- to 8-week-old C57BL/6J mice injected with Ad-uc.372m or Ad-NCm for 7 days (*n* = 3). **e** Expression levels of genes related to lipid synthesis, uptake, oxidation, and secretion in HepG2 cells transfected with Ad-uc.372i or Ad-NCi for 48 h (*n* = 3). **f** Protein levels of ACC, FAS, SCD1, and CD36 in HepG2 cells transfected with Ad-uc.372i or Ad-NCi for 48 h in the presence of an O/P mixture (representative blots from three similar experiments) (*n* = 3). **g** Expression levels of *acc*, *fas*, *scd1*, and *cd36* in the liver of 10-week-HFD-fed mice after tail vein injection of Ad-uc.372i or Ad-NC for 7 days (*n* = 3). **h** Protein levels of acc, fas, scd1, and cd36 in the liver of 10-week-HFD-fed mice after tail vein injection of Ad-uc.372i or Ad-NC for 7 days (*n* = 3). Data are mean ± SEM; *P < 0.05; **P < 0.01; ***P < 0.001 vs. control group (Student’s *t*-test)

To explore the underlying mechanism by which uc.372 regulates hepatic lipid metabolism, we assessed the expression levels of genes related to lipid synthesis (*ACC*, *FAS*, *SCD*, *SREBP1*, and *LXR*), uptake (*Fatp1*, *Fatp2*, *Fatp5*, *Fabp1*, and *CD36*), oxidation (*Cpt1a, Scad*, *Acox1*, and *PPARα*) and secretion (*apoB* and *Mtp*) in HepG2 cells transfected with Ad-uc.372m. We found that expression of genes related to lipid synthesis, including *ACC*, *FAS*, and *SCD1*, and genes related to lipid uptake, such as *CD36*, were upregulated upon HepG2 cells overexpressing uc.372 compared to control cells (Fig. 3a, b and Supplementary Fig. 3a). Similarly, overexpression of uc.372 in the livers of C57BL/6J mice led to elevated levels of acc, fas, scd1, and cd36 (Fig. 3c, d, and Supplementary Fig. 3b).

In contrast, suppression of uc.372 expression by transfection of Ad-uc.372i into HepG2 cells pre-treated with an O/P mixture resulted in much lower mRNA and protein levels of ACC, FAS, SCD1, and CD36 compared to control cells (Fig. 3e, f and Supplementary Fig. 3c). Moreover, silencing of uc.372 in the liver of HFD-fed mice reduced expression of acc, fas, scd1, and CD36 (Fig. 3g, h and Supplementary Fig. 3d). These results reveal that uc.372 drives hepatic steatosis by promoting de novo lipogenesis and lipid uptake.

### uc.372 inhibits the maturation of miR-195/miR-4668

**Fig. 4:**
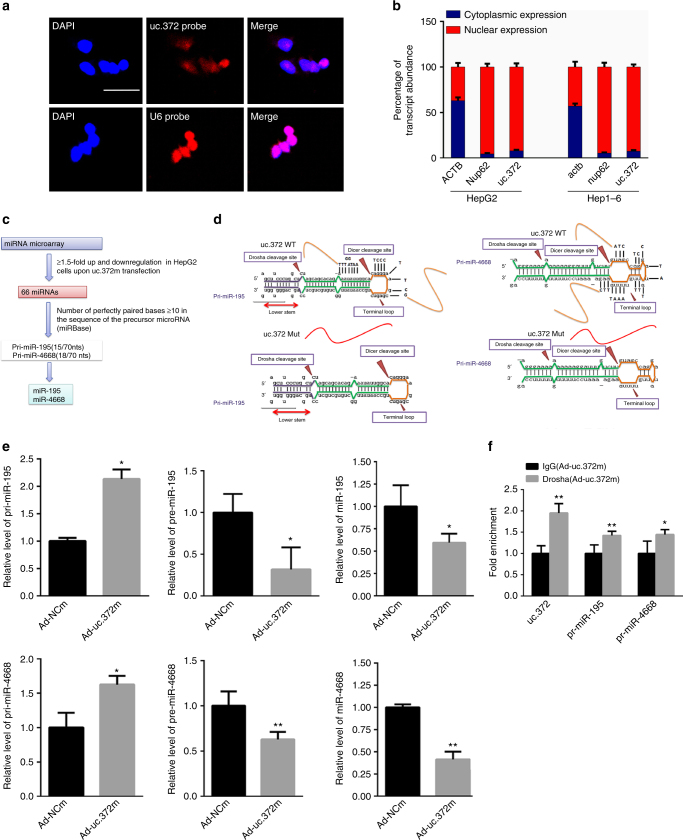
uc.372 binds to pri-miR-195/pri-miR-4668. **a** Representative image from three similar experiments showed the distribution of uc.372 in HepG2 cells as analyzed by in situ hybridization. Scale bar, 25 μm. **b** Cellular fractionation assay in HepG2 and Hep1-6 cells was performed by quantitative real-time PCR using a specific cytosol control (gene *ACTB*) and a specific nuclear control (gene *Nup62*) (*n* = 3). **c** Schematic flowchart depicting the strategy. **d** The stem-loop sequence of pri-miR-195 (left panel) and pri-miR-4668 (right panel), and their partial complementarity with uc.372. **e** The levels of pri-miR-195/pri-miR-4668, pre-miR-195/pre-miR-4668, and mature miR-195/miR-4668 in the HepG2 cells transfected with Ad-uc.372m or Ad-NCm for 48 h (*n* = 3). **f** RIP assay showed that uc.372 could bind pri-miR-195/pri-miR-4668 in HepG2 cells transfected with Ad-uc.372m or Ad-NCm for 48 h (*n* = 3). Data are mean ± SEM; *P < 0.05; **P < 0.01 vs. control group (Student’s *t*-test)

To understand the mechanism by which uc.372 regulates the expression of ACC, FAS, SCD1, and CD36, we analyzed uc.372 distribution in HepG2 cells using in situ hybridization. In HepG2 cells, uc.372 was predominantly localized to the nucleus (Fig. 4a). The nuclear/cytoplasmic ratio of uc.372 expression in human HepG2 cells and Hep1-6 cells was 12.6 and 13.3, respectively (Fig. 4b), suggesting that uc.372 might exert its biological function in the nucleus. The correlation between miRNAs and UCEs has been widely reported^20,24,25^, indicating possible regulatory mechanisms that could connect both types of ncRNAs. To identify putative miRNA targets for uc.372, we overexpressed uc.372 in HepG2 cells and carried out a microarray analysis. A total of 66 miRNAs present in the array demonstrated ≥1.5-fold upregulation or downregulation of expression upon uc.372 transfection (Fig. 4c, Supplementary Fig. 4a, 4b, and 4c). Of note, pri-miR-195 and pri-miR-4668 displayed a complementarity with the ultraconserved region of uc.372. This complementarity involved nucleotides located at the terminal loop region site within the miR-195 and miR-4668 primary transcripts (Fig. 4d).

In the nucleus, pri-miRNAs undergo Drosha-dependent cleavage into pre-miRNAs before being exported to the cytoplasm^22^. To explore the interaction between miR-195/miR-4668 and uc.372, we determined expression levels of pri-miR-195/pri-miR-4668, pre-miR-195/pre-miR-4668, and miR-195/miR-4668 upon uc.372 inhibition or overexpression. Of note, significantly reduced pri-miR-195/pri-miR-4668 and elevated pre-miR-195/pre-miR-4668 and mature miR-195/miR-4668 were observed in the HepG2 cells transfected with Ad-372i (Supplementary Fig. 5a). By contrast, overexpression of uc.372 in the HepG2 increased the levels of pri-miR-195/pri-miR-4668 and repressed the maturation of miR-195/miR-4668 (Fig. 4e). To disrupt the interaction between uc.372 and these miRNAs, we constructed mutants in which the complementary nucleotides were deleted from uc.372 (Fig. 4d). As expected, when this mutant form of uc.372 was overexpressed, no changes in pri-miR-195/pri-miR-4668, pre-miR-195/pre-miR-4668, and mature miR-195/miR-4668 expression were identified (Supplementary Fig. 5b). To further assess that pri-miR-195/pri-miR-4668 interacts with uc.372, RNA-binding protein immunoprecipitation (RIP) assay was performed with an antibody against Drosha, a key protein in the processing of pri-miRNA into pre-miRNA. As shown in Fig. 4f, overexpression of uc.372 significantly increased the level of pri-miR-195/pri-miR-4668 in the immunoprecipitated RNA with antibody against Drosha, indicating a binding between uc.372 and the terminal loop region of pri-miR-195/pri-miR-4668.

**Fig. 5:**
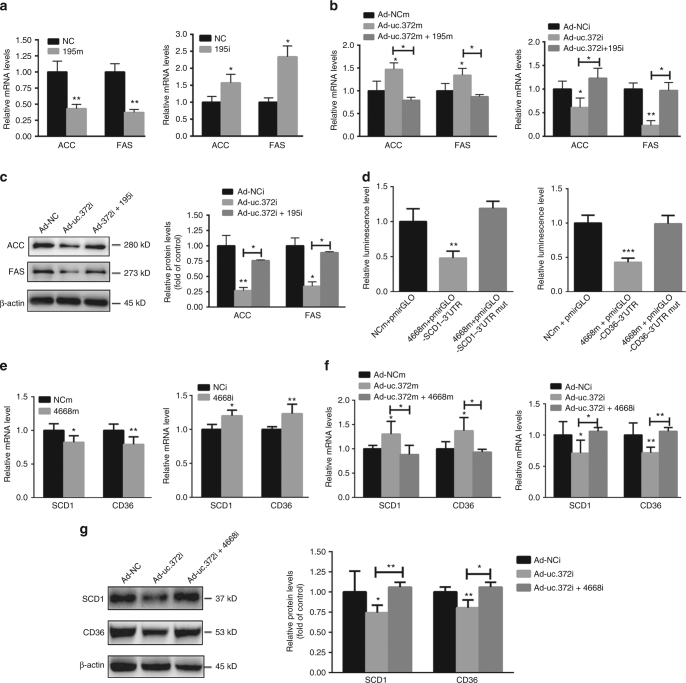
uc.372 inhibits the maturation of miR-195/miR-4668 to regulate expression of ACC, FAS, SCD1, and CD36. **a** Expression levels of *ACC* and *FAS* in the HepG2 cells transfected with miR-195 mimic and inhibitor at a final concentration of 20 nM for 48 h (*n* = 3). **b** Expression levels of *ACC* and *FAS* in the HepG2 cells transfected with miR-195 mimic and inhibitor for 24 h in the presence with Ad-uc.372m and Ad-uc.372i (*n* = 3). **c** Protein levels of ACC and FAS in Ad-uc.372i-infected and miR-195 inhibitor-transfected HepG2 cells in the presence with with 300 μM O/P mixture for 48 h (representative blots from three similar experiments) (*n* = 3). **d** The relative luciferase units (RLU) in the HepG2 cells transfected with pmirGLO-SCD1-3′UTR and pmirGLO-SCD1-3′UTR mutant or pmirGLO-CD36-3′UTR and pmirGLO-CD36-3′UTR mutant under overexpression of miR-4668 or NC for 48 h (*n* = 3). **e** Expression levels of *SCD1* and *CD36* in the HepG2 cells transfected with miR-4668 mimic and inhibitor or NC for 48 h (*n* = 3). **f** Expression levels of *SCD1* and *CD36* in the HepG2 cells transfected with miR-4668 mimic and inhibitor for 24 h in the presence with Ad-uc.372m and Ad-uc.372i (*n* = 3). **g** Protein levels of ACC and FAS in Ad-uc.372i-infected and miR-4668 inhibitor-transfected HepG2 cells in the presence with 300 μM O/P mixture for 48 h (representative blots from three similar experiments) (*n* = 3). Data are mean ± SEM; *P < 0.05; **P < 0.01; ***P < 0.001 vs. control group (**a**, **e** Student’s *t*-test; **b**, **c**, **d**, **f**, **g** analysis of variance (ANOVA))

Given the direct RNA–RNA interaction, we assessed whether uc.372 affected lipid metabolism through miR-195/miR-4668-mediated target genes. Previous studies have shown that *ACC* and *FAS* are target genes of miR-195^26^ (Supplementary Fig. 6a). Based on the reduced levels of miR-195 in HepG2 cells treated with an O/P mixture (Supplementary Fig. 6a), we investigated the effects of miR-195 on expression of ACC and FAS. As shown by real-time PCR analysis, miR-195 markedly suppressed mRNA levels of FAS and ACC, even when uc.372 was overexpressed in HepG2 cells (Fig. 5a, b). More importantly, the uc.372 inhibition-dependent reduction in ACC and FAS expression and impaired intracellular triglyceride contents could be largely restored by miR-195 suppression (Fig. 5c, Supplementary Fig. 6b, Supplementary Fig. 7a, b), suggesting that reduced miR-195 suppression of ACC and FAS expression plays a key role in uc.372-induced lipid accumulation.

Based on TargetScan, *SCD1* and *CD36* were predicted as possible target genes of miR-4668 (Supplementary Fig. 6c). We determined expression of miR-4668 in HepG2 cells treated with an O/P mixture, and observed decreased expression of miR-4668 in the O/P -treated HepG2 cells compared to controls (Supplementary Fig. 6c). To verify that *SCD1* and *CD36* are true targets of miR-4668, the 3′ untranslated regions (UTRs) of these genes containing the binding sites for miR-4668 were cloned into the pmirGLO plasmid to assess luciferase activity. As shown in Fig. 5d, miR-4668 significantly suppressed the relative luciferase units of pmirGLO-SCD1-3′UTR or pmirGLO-CD36-3′UTR, whereas these affects were rescued by expression of pmirGLO-SCD1-3′UTR pmirGLO-CD36-3′UTR mutants lacking the miR-4668-binding site. Further studies revealed that miR-4668 reduced the mRNA levels of SCD1 and CD36 even when uc.372 was overexpressed in HepG2 cells (Fig. 5e, f). Conversely, the uc.372 silencing-dependent reduction in SCD1 and CD36 expression and lipid accumulation could be partially rescued by miR-4668 inhibition (Fig. 5g, Supplementary Fig. 6d, Supplementary Fig. 7c, d).

Taken together, these data demonstrate that uc.372 specifically suppresses miR-195/miR-4668 expression by binding to pri-miR-195/pri-miR-4668 to relieve miR-195/miR-4668-mediated suppression of functional target genes such as *ACC*, *FAS*, *SCD1*, and *CD36*, leading to lipid accumulation in hepatocytes.

### *INSM2* may be involved in the transcription of uc.372

**Fig. 6:**
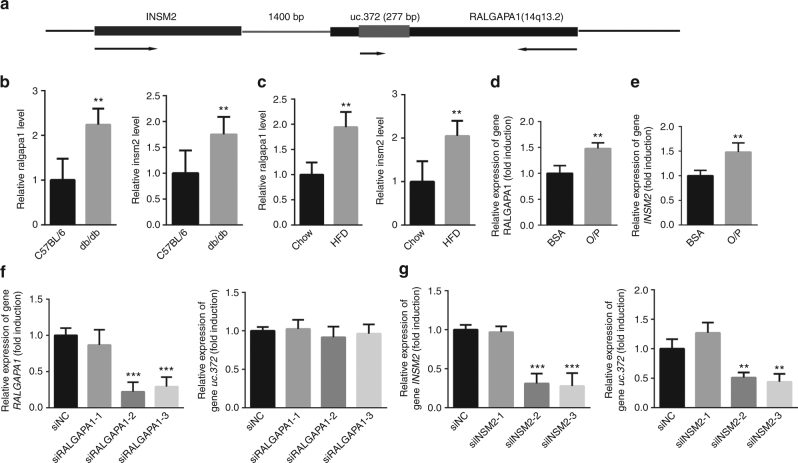
uc.372 expression is dependent on transcription of INSM2. **a** Schematic analysis of uc.372 location. **b** *Ralgapa1* and *insm2* expression in the liver of 8-week-old male db/db mice (*n* = 5). **c** *Ralgapa1* and *insm2* expression in the liver of 10-week-HFD-fed mice (*n* = 5). **d** The mRNA level of *RALGAPA1* in the HepG2 cells treated with 300 μM O/P mixture for 48 h (*n* = 3). **e** The mRNA level of *INSM2* in the HepG2 cells treated with 300 μM O/P mixture for 48 h (*n* = 3). **f** The levels of *RALGAPA1* and uc.372 after silencing *RALGAPA1* in HepG2 cells for 48 h (*n* = 3). **g** The levels of *INSM2* and uc.372 after silencing *INSM2* in HepG2 cells for 48 h (*n* = 3). Data are mean ± SEM; **P < 0.01; ***P < 0.001 vs. control group (**b**, **c**, **d**, **e** Student’s *t*-test; **f**, **g** analysis of variance (ANOVA))

We next sought to understand how the transcription of uc.372 is regulated. By analyzing the sequence of uc.372, we found that it consists of 277 nucleotides (nt) that are highly conserved across species. The uc.372 ultraconserved region is located within the intronic region of the *RALGAPA1* gene and is also located 1400 bp downstream of the insulinoma-associated 2 gene (*INSM2*) on chromosome 14 (Fig. 6a). To explore the possible interrelationships between *RALGAPA1*,* INSM2*, and uc.372 transcription, the mRNA levels of *RALGAPA1* and *INSM2* were first evaluated by real-time PCR in the livers of db/db mice and HFD-fed mice. The results indicated that the expression of both *RALGAPA1* and *INSM2* was increased in the livers of these animal models (Fig. 6b, c). In HepG2 cells treated with a 300 μM O/P mixture, the mRNA levels of *RALGAPA1* and *INSM2* were also enhanced (Fig. 6d, e). Furthermore, no changes in uc.372 expression were identified in HepG2 cells transfected with siRNA against *RALGAPA1*, despite a 71–78% decrease in *RALGAPA1* mRNA levels (Fig. 6f). However, uc.372 expression was significantly downregulated in HepG2 cells treated with siRNAs targeting *INSM2* (Fig. 6g). These observations suggest that *INSM2* may be involved in the transcriptional regulation of uc.372.

### USF1 transcriptionally regulates INSM2 and uc.372 expression

**Fig. 7:**
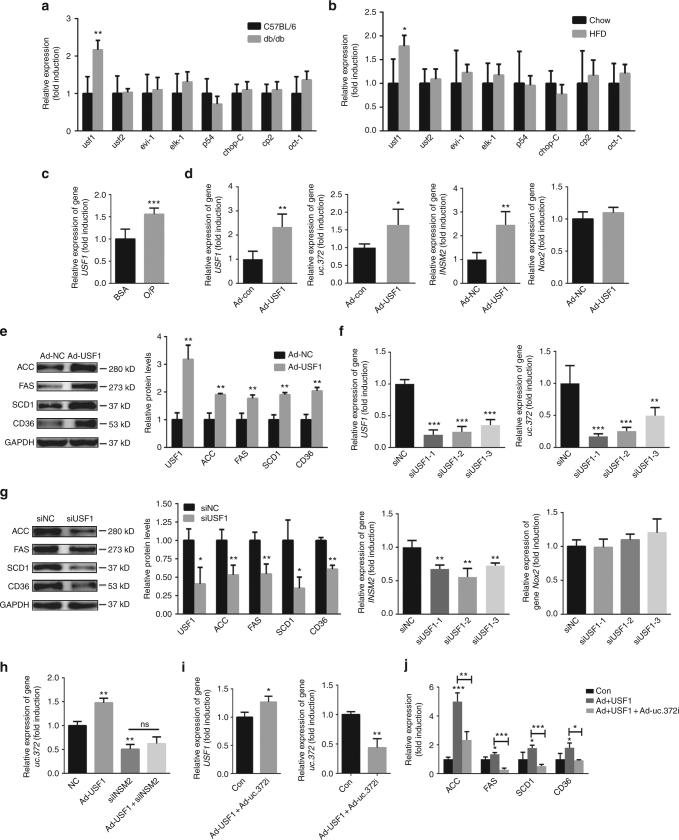
Upstream transcription factor 1 (USF1) transcriptionally regulates expression of INSM2 and uc.372. **a** Identification of *usf1* elevated in the liver of 8-week-old male db/db mice (*n* = 5). **b** Identification of *usf1* elevated in the liver of 10-week-HFD-fed mice (*n* = 5). **c** Expression of *USF1* in the HepG2 cells treated with 300 μM O/P mixture for 48 h (*n* = 3). **d** Expression levels of *USF1*, uc.372, *INSM2*, and *Nox2* in the HepG2 cells infected with Ad-USF1 or Ad-NC for 48 h (*n* = 3). **e** Protein levels of ACC, FAS, SCD1, and CD36 in the HepG2 cells infected with Ad-USF1 or Ad-NC for 48 h (representative blots from three similar experiments) (*n* = 3). **f** Real-time PCR analysis of *USF1*, uc.372, *INSM2*, and *Nox2* in the HepG2 cells transfected with specific siRNAs targeting *USF1* or *NC* for 48 h (*n* = 3). **g** Western blot analysis of ACC, FAS, SCD1, and CD36 expression in the HepG2 cells transfected with specific siRNAs targeting USF1 (representative blots from three similar experiments) (*n* = 3). **h** The mRNA level of uc.372 in the HepG2 cells infected with Ad-USF1 in the presence or absence of siINSM2 or NC for 48 h (*n* = 3). **i** The mRNA levels of *USF1* and uc.372 in the HepG2 cells infected with Ad-USF1 and Ad-uc.372i for 48 h (*n* = 3). **j** The mRNA levels of *ACC*, *FAS*, *SCD1*, and *CD36* in the HepG2 cells infected with Ad-USF1 and Ad-uc.372i or Ad-NC for 48 h (*n* = 3). Data are mean ± SEM; *P < 0.05; **P < 0.01; ***P < 0.001 vs. control group. (**a**, **b**, **c**, **d**, **e**, **g**, **i** Student’s *t*-test; **f**, **h**, **j** analysis of variance (ANOVA))

Given that transcription of uc.372 is dependent on *INSM2*, we proceeded to explore the transcription factors modulating this process. Six transcription factors targeting uc.372 were identified through the Gene Regulation database (http://www.gene-regulation.com/pub/databases.html). As shown by real-time PCR analysis, the expression of upstream transcription factor 1 (*USF1*) was elevated in the livers of both db/db and HFD-fed mice (Fig. 7a, b). We also observed a significantly increased level of *USF1* in HepG2 cells treated with an O/P mixture (Fig. 7c). The consistent expression patterns of *USF1* with *INSM2* raised the possibility that *USF1* could transcriptionally regulate expression of *INSM2*. To further test this hypothesis, *USF1* was overexpressed in HepG2 cells by adenovirus transduction. Compared to control cells, cells overexpressing *USF1* showed markedly enhanced mRNA levels of uc.372 and *INSM2*, but not the negative control of *NOX2* (Fig. 7d). In addition, overexpression of USF1 also resulted in a significant increase in the protein levels of ACC, FAS, SCD1, and CD36 (Fig. 7e, Supplementary Fig. 8a). In contrast, decreasing USF1 expression reduced the mRNA levels of uc.372 and *INSM2*, but not the negative control of *NOX2* (Fig. 7f) and the protein levels of ACC, FAS, SCD1, and CD36 (Fig. 7g, Supplementary Fig. 8b). Importantly, overexpression of *USF1* failed to enhance uc.372 expression in HepG2 cells with knockdown of *INSM2* (Fig. 7h), indicating that direct or indirect regulation of *INSM2* on uc.372 may exist since *INSM2* is a well-characterized transcriptional repressor 2^27^. To further verify that *USF1* exerts its functional role through regulation of uc.372, we employed a rescue strategy in HepG2 cells. Inhibition of uc.372 significantly blunted the *USF1*-mediated induction of *ACC*, *FAS*, *SCD1*, and *CD36* expression (Fig. 7I, j). Based on these data, we propose that *USF1* transcriptionally regulates the expression of *INSM2* and uc.372, thereby elevating the expression of *ACC*, *FAS*, *SCD1*, and *CD36* in hepatocytes.

### The effect of uc.372 was validated in NAFLD patients’ livers

**Fig. 8:**
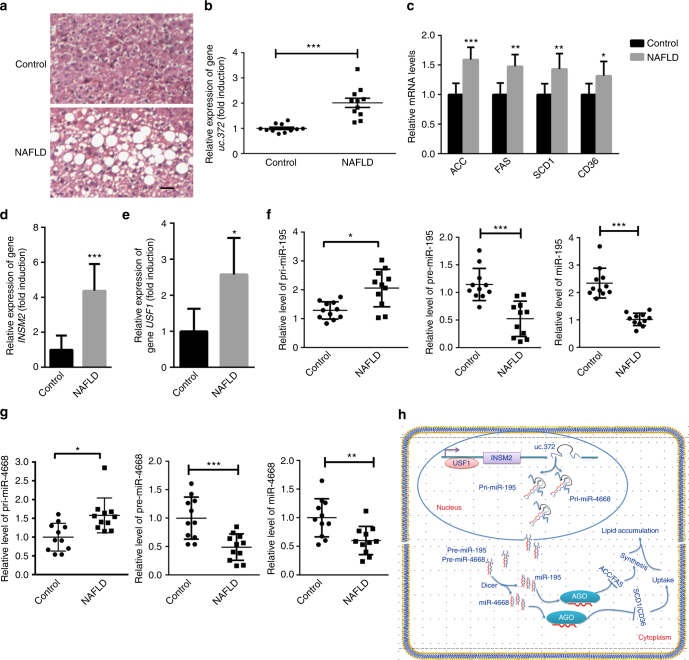
Effect of uc.372 on abnormal lipid accumulation is subjected to regulation of miR-195/ miR-4668 in the liver of NAFLD patients. **a** Oil Red O staining of liver frozen sections from NAFLD patients or healthy control (representative image from three similar experiments). Scale bar, 100 μm. **b** The level of uc.372 in the liver samples of NAFLD patients or healthy control (*n* = 11). **c** The mRNA levels of *ACC*, *FAS*, *SCD1*, and *CD36* in the liver samples of NAFLD patients or healthy control (*n* = 3). **d** The mRNA level of *INSM2* in the liver samples of NAFLD patients or healthy control (*n* = 11). **e** The mRNA level of *USF1* in the liver samples of NAFLD patients or healthy control (*n* = 11). **f** The levels of pri-miR-195, pre-miR-195, and mature miR-195 in the liver samples of NAFLD patients or healthy control (*n* = 11). **g** The levels of pri-miR-4668, pre-miR-4668, and mature miR-4668 in the liver samples of NAFLD patients or healthy control (*n* = 11). **h** Schematic depicting our proposed model that us.372 drives hepatic lipid accumulation. Data are mean ± SEM; Student’s *t*-test: *P < 0.05; **P < 0.01; ***P < 0.001 vs. control group

We next asked whether uc.372 was functionally involved in the abnormal hepatic lipid accumulation of NAFLD patients by regulation of pri-miR-195/pri-miR-4668 processing. In addition to abnormal lipid accumulation, we also observed a marked upregulation of hepatic uc.372 expression in NAFLD patients (Fig. 8a, b). Microarray transcriptome profiling analysis revealed that lipid metabolism pathways and uc.372 expression were aberrantly changed in the liver of NAFLD patients (Supplementary Fig. 9a, b). This prompted us to further examine genes related to lipogenesis and lipid uptake using real-time PCR. We confirmed that the levels of *ACC*, *FAS*, *SCD1*, and *CD36* were significantly elevated in the liver of NAFLD patients (Fig. 8c). Congruently, we found significant enhancement of hepatic *INSM2* and *USF1* expression, supporting the idea that *USF1* transcriptionally regulates *INSM2* and uc.372 expression in the liver of NAFLD patients (Fig. 8d, e). Subsequently, we analyzed the hepatic levels of pri-miR-195/pri-miR-4668 and mature miR-195/miR-4668 in these patients. Intriguingly, we observed an increase of pri-miR-195/pri-miR-4668 and a reduction of miR-195/miR-4668 expression in the liver of NAFLD patients (Fig. 8f, g). These results confirm that the lipid accumulation effect of uc.372 is subject to regulation by miR-195/miR-4668 in the steatotic liver of NAFLD patients.

## Discussion

Recently, ucRNAs have been identified as a class of highly conserved lncRNAs, but a clear understanding of the physiological significance and potential pathological roles of ucRNAs has remained elusive. Much of the research on ucRNAs has focused on elucidating their evolutionary significance^28,29^ and the tumor oncogenic or suppressive roles of these ucRNAs in human cancers^20,21,23^. Moreover, emerging reports indicate that lncRNAs are key regulators of lipid metabolism and fatty liver disease^30,31^. For example, lncLSTR, a liver-enriched lncRNA, regulates a TDP-43/FXR/ApoC2-dependent pathway to maintain lipid metabolism in mice^32^. However, the role of ucRNAs in lipid metabolism and pathogenesis of NAFLD remained unclear. In the present study, we identify the upregulation of uc.372 in the livers of db/db and HFD-fed mice and NAFLD patients. It is very interesting that the expression of uc.348, 372, 94, 157, and 436 was dramatically increased in the liver of db/db mice, but not in C57 mice at 10 weeks after HFD administration. To analyze the reason for this inconsistence, we further examined expression of uc.348, 372, 94, 157, and 436 in the liver of C57 mice at 5, 10, 15, and 22 weeks after HFD administration and the liver of NAFLD patients. The level of uc.372 was continuously increased in the liver of C57 mice at 5, 10, 15, and 22 weeks, whereas the levels of uc.348 and 94 were increased at 15 or 22 weeks after HFD administration. However, no changes of uc.157 and 436 were identified (Supplementary Fig. 1d). Moreover, in the liver of NAFLD patients, the expression levels of uc.372 and uc.157 were increased, but that of uc.94 was decreased (Supplementary Fig. 9c). These results indicated that this inconsistence might be due to different pathogeneses or pathological stages. The liver is well established as the most important organ for the biogenesis of major metabolites such as lipids^33^. In the liver, aberrant hepatic fat deposits result in the development of NAFLD^34^. Our data demonstrate an important contribution of hepatic uc.372 to the impaired homeostasis of lipid metabolism and implicate it as a previously uncharacterized lncRNA that plays a critical role in the pathogenesis of NAFLD. It is noteworthy that in the HFD-fed mice, the expression of uc.372 was dramatically increased in the muscle and fat, the organs also closely participating in NAFLD progression due to their critical roles in lipid metabolism and insulin resistance. To assess whether the regulation of hepatic steatosis by uc.372 is a direct effect on hepatocytes or derives from its influence on fat or muscle, for instance, the lipolysis of adipocytes, we examined the expression of green fluorescent protein (GFP) and uc.372 in different tissues after tail vein injection with Ad-uc.372m. After injection, GFP was mainly expressed in the liver of mice (Supplementary Fig. 2g). Similarly, Ad-uc.372m injection led to dramatically increased uc.372 in the liver, but not in other tissues (Supplementary Fig. 2h). These data suggest that overexpressed hepatic uc.372 directly induced hepatic steatosis. So, what is the effect of uc.372 on the lipid metabolism in adipose tissue and muscle? Given the importance of skeletal muscle to our overall physical and metabolic capacities^35^, improving our understanding of the changes to muscle health during the development of obese and insulin resistant states will advance the development of therapeutic strategies for those individuals. While the exact cause of muscle metabolism disorders with the development of diet-induced obesity is still unknown, one proposed mechanism is elevated delivery of free fatty acids (FFAs) in conjunction with impaired lipid metabolism within skeletal muscle leading to a build-up of intramyocellular lipids and associated lipid derivatives^36^. Recent advances have shown that lncRNAs are associated with skeletal muscle development, growth, and function^37–39^. For instance, H19, malat1, MyoD upstream lncRNA, lncMyoD, developmental pluripotency-associated 2 upstream binding muscle lncRNA, and Linc-MD1^37–40^ are all regulated during myogenesis, and likely necessary for efficient differentiation. However, little study has focused on the role of lncRNAs in the lipid metabolism of muscle tissues in HFD mice. In the present study, we found the level of uc.372 was increased in the muscle tissues of HFD mice compared with that of control. Moreover, pre-incubation with O/P significantly enhanced the level of uc.372 in the liver cells. Therefore, we propose that the elevated FFAs level in muscle may induce the expression of uc.372, which is then involved in the progression of HFD-induced metabolic disorders. In the future, it is necessary to explore the role of uc.372 in fat or muscle, for instance, the lipolysis of adipocytes, by using fat or muscle-specific uc.372-TG and KO mice.

It has been demonstrated that multiple factors are involved in hepatic fat accumulation, including enhanced de novo lipogenesis and absorption as well as decreased fatty acid oxidation and secretion^41^. To provide further insights to the role of uc.372 in hepatic lipid metabolism, we analyzed the expression of genes related to lipid metabolism in vivo and in vitro. We found that uc.372 regulated expression of genes related to lipid synthesis, including *ACC*, *FAS*, and *SCD1*, and genes related to lipid uptake, such as *CD36*, suggesting that uc.372 drives hepatic lipid accumulation through the promotion of de novo lipogenesis and lipid uptake.

We next sought to understand how uc.372 regulates expression of ACC, FAS, SCD1, and CD36. Of note, intronic ultraconserved regions are frequently related to the regulation of transcription and DNA binding, indicating a potential role as regulators of gene expression^16^. Given the structural similarity of miRNAs and lncRNAs, miRNAs may also bind to lncRNA sequences. For instance, multiple lncRNAs have been identified as competing endogenous RNAs (ceRNAs)^42^. In general, ceRNAs function as miRNA sponges and decrease the activity of target miRNAs without changing their biogenesis^43,44^. Recent studies have demonstrated lncRNA-mediated regulatory networks for miRNA processing^20,22^. For example, lower-stem strand invasion by uc.283 + A impairs microprocessor recognition and efficient pri-miR-195 cropping^22^. The processing of pri-miRNA into pre-miRNA requires direct interaction with the Drosha-DGCR8 microprocessor complex to modulate miRNA maturation^45,46^. The pri-miRNA/pre-miRNA hairpin structure consists of mismatches, internal loops, and bulges^47^. The terminal loop of these hairpins demonstrates a variable structure, which may be important for the rate at which a specific miRNA is processed^48^. It is suggested that a flexible terminal loop plays a key role in processing by Drosha, as decreasing the length or changing the sequence of a terminal loop significantly influences pri-miRNA processing efficiency^49,50^. In situ hybridization and cytoplasmic and nuclear fractioning demonstrates that uc.372 is mainly located in the nucleus of HepG2 cells. The nuclear localization of uc.372 suggests it may bind to mRNA to modulate gene transcription. Further studies by RIP assay revealed that pri-miR-195 and pri-miR-4668 interacts with the ultraconserved region of uc.372 at the terminal loop region site within the miR-195 and miR-4668 primary transcript. More importantly, uc.372 regulates pri-to-pre-miRNA cleavage of miR-195 and 4668 by binding pri-miR-195/pri-miR-4668.

Abnormal expression of miR-195 has been widely reported in diverse biological systems^51,52^. For instance, miR-195 can bind the 3′UTRs of *cyclin D1*,* CDK6*, and *E2F3*, thereby suppressing cell proliferation in human hepatocellular carcinoma cells^52^. Additionally, miR-195 suppresses cell proliferation, invasion, and metastasis in breast cancer cells by targeting *FAS*, *HMGCR*, *ACC*, and *CYP27B1*^26^. Consistently, we found that miR-195 markedly suppresses mRNA levels of *FAS* and *ACC*, even when uc.372 is overexpressed in HepG2 cells. However, less is known about the functional role of miR-4668. Our results suggest that uc.372 suppresses miR-4668 expression through binding pri-miR-4668 to relieve miR-4668-mediated suppression of functional target genes such as *SCD1* and *CD36*.

How miR-195/miR-4668 link to the metabolic genes such as *FAS*, *ACC*, *SCD1*, and *CD36*? It was showed that once miRNAs processed from the hairpin and loaded into the Ago2 of the silencing complex, the miRNAs pair with mRNAs to direct posttranscriptional repression^53^. In animals, the canonical model posits that the 22- to 23-nt miRNAs guide Ago2 to target sites in mRNA 3′UTR and coding sequences through imperfect sequence complementarity. Complementarity in the “seed” region of the miRNA, nt 2–8, is a key determinant of the interaction^54^. At sites with extensive pairing complementarity, metazoan miRNA can direct Argonaute-catalyzed mRNA cleavage^53,55^. In the present study, the sequence alignment results in Supplementary Fig. 6a, b showed canonical complementarity between miR-195/miR-4668 and their respective targets, which is necessary for the formation of the miRNA–mRNA duplex^56^. And dual luciferase reporter assay suggested that miR-195/miR-4668 could reduce the relative luciferase activity. Based on the previous studies and our observations, we consider that *FAS* and *ACC/SCD1* and *CD36* are direct targets of miR-195/miR-4668 mediated by Ago2.

Finally, we identify *USF1* as transcription factor that regulates uc.372 expression. Through the Gene Regulation database, we found that the sequence of uc.372 overlaps with that of *RALGAPA1* and is located downstream of *INSM2*, which plays a key role in insulin islet development^27^. Further studies revealed that the transcription of *RALGAPA1* and uc.372 is independent. However, our data showed that knockdown of *INSM2* suppressed the level of uc.372 and the regulation of USF1 on uc.372. How *INSM2* mediates regulation of *USF1* on uc.372? If uc.372 shares the promoter with *INSM2*, the regulation on each should be independent of the other. We propose that direct or indirect regulation of *INSM2* on uc.372 may exist since *INSM2* is a well-characterized transcriptional repressor 2^27^. Therefore, further study is necessary to elucidate the potential mechanism underlying the regulation of uc.372. In the present study, we identify *USF1*, which has been shown to participate in the transcriptional regulation of several lipid metabolism-related genes^57,58^, as a major transcription factor regulating the expression of *INSM2* and uc.372, thereby mediating uc.372-induced hepatic lipid accumulation.

In summary, as shown in Fig. 8h, our findings reveal a novel mechanism by which uc.372 drives hepatic steatosis by repressing the maturation of miR-195/miR-4668, thus relieving the suppression of target genes, including *ACC*, *FAS*, *SCD1*, and *CD36*. We also propose that uc.372 inhibitors might be potential therapeutic agents for NAFLD. In addition, we demonstrate that the expression of uc.372 is regulated by *USF1* and is dependent on the transcription of *INSM2*. Of note, an analysis of uc.372 distribution in various tissues identifies abundant uc.372 expression in metabolism-related organs of HFD-fed mice, including muscle, liver, heart, and fat. Therefore, a main objective for the future would be to investigate the potential functional roles of this lncRNA in different metabolism-related organs to maintain lipid homeostasis.

## Methods

### Experimental animals

The animals used in the experiment including 8-week-old male db/db mice (*n* = 6) and age-matched male WT C57BL/6J mice (*n* = 6; Peking University Health Science Center, Beijing, China). These mice were originally obtained from the Jackson Laboratory and fed a standard chow diet for 4 weeks. To establish the NAFLD model, 5-week-old male C57BL/6J mice were given free access to a standard chow diet or a HFD (D12451, 45% kcal from fat, Research Diet, USA, http://www.researchdiets.com/opensource-diets/stock-diets) for 10 weeks in a temperature (20–24 °C)- and humidity-controlled (45–55%) environment with a 12 h/12 h light/dark cycle. Tail vein injection was carried out in HFD-fed mice with an adenoviral vector expressing uc.372 inhibitor (Ad-uc.372i) or negative control adenoviral vector (Ad-NCi). Furthermore, 6- to 8-week-old male C57BL/6 J mice fed a standard chow diet were transfected with the adenoviral vector expressing uc.372 mimic (Ad-uc.372 m) or negative control adenoviral vector (Ad-NCm) through tail vein injection. On day 7 after adenoviral vector injection, the mice were anesthetized, and blood was collected via cardiac puncture. Livers were harvested, snap-frozen in liquid nitrogen, and stored at −80 °C for further analysis^59^.

This mouse research procedures followed the guidelines of the Animal Ethics Committee at Beijing Hospital (BJMOH-201402).

### Human liver specimens

Human liver biopsies from 10 patients with NAFLD and 10 healthy control subjects were performed with patient consent within the diagnostic workup of NAFLD. The application for patient-derived materials was approved by the Research Ethics Committee of Beijing You-An Hospital (BJYSE2015-35), and written consent was obtained from all patients. Clinical and biochemical characteristics of healthy individual and patients with NAFLD were analyzed^60^.

### miRNA array analysis

Total 15 mg RNA was isolated from the mixed samples of liver tissues from five male db/db mice and five age-matched male WT mice or liver tissues from five NAFLD patients and five healthy controls. Total RNA was reverse-transcribed with biotin end-labeled random oligonucleotide primers. The transcsriptome for mouse lncRNAs was analyzed by the Agilent mouse lncRNA + mRNA Array V1.0 (CapitalBio; Beijing, China), while that for human lncRNAs was analyzed by Agilent human lncRNA + mRNA Array V4.0 (CapitalBio), including sense and antisense probes to all 481 human ultraconserved sequences, each spotted in duplicate. For RNA isolated from HepG2 cells, the transcriptome for human miRNA was analyzed using Agilent human miRNA Microarray V21.0 (CapitalBio).

The total RNA samples were Cy3-labeled with CapitalBio cRNA Amplification and Labeling Kit. And the miRNA samples were Cy3-labeled using the Agilent miRNA Complete Labeling and Hyb Kit, according to the manufacturer’s guidelines. In strict accordance with the standard pipeline, the microarray hybridization, washing, and scanning were carried out. To analyze the array images, Agilent feature extraction software version 10.10 was applied. The GeneSpring GX software (Agilent Technologies) was used for normalization and subsequent data processing. The differentially expressed lncRNA or miRNSs were obtained using threshold values of ≥1.5- and ≤−1.5-fold change and a *t*-test *p* value of 0.05 for all transcriptome comparisons.

### Cell culture

Human hepatic carcinoma cell line HepG2 derived from a liver hepatocellular carcinoma of a 15-year-old Caucasian male and murine hepatic cancer cell line Hep1-6 derived from the BW7756 mouse hepatoma that arose in a C57/L mouse were purchased from American Type Culture Collection. Both cells were cultured in Eagle’s minimum essential medium (MEM; Invitrogen, Carlsbad, CA, USA) and supplemented with 10% fetal bovine serum (FBS; HyClone, Logan, UT, USA), 100 U/ml penicillin (Invitrogen), and 0.1 mg/ml streptomycin (HyClone) at 37 °C with humidified air and 5% CO_2_.

### Construction of adenoviral vectors

Adenoviral vectors containing uc.372 mimic (Ad-uc.372m), USF1 (Ad-USF1), uc.372 inhibitor (Ad-uc.372i), and control vector (Ad-NC) were constructed by Genepharma (Shanghai, China). Two shRNAs (GGAGGGATGATCCAATCTAAT and GGCAGATTGTTCTAAGTGATA) against uc.372 were selected to control for off-target effects.

### Transient transfection

*INSM2* siRNA, *RALGAPA1* siRNA, *USF1* siRNA, and their nonspecific siRNA (NC) were constructed by Genepharma. Transfection of siRNAs was performed with HiPerFect transfection reagent (Qiagen, Duesseldorf, Germany) as previously described^61^. Briefly, 6 × 10^5^ cells were seeded in six-well plates with 2 ml MEM culture medium containing 10% FBS and antibiotics. At the same time, siRNAs or NC were mixed with HiPerFect transfection reagent and incubated at room temperature for 10 min. Then, the complexes were transfected into HepG2 cells for 48 h.

### RNA isolation and real-time PCR

Total RNA from HepG2 cells was extracted with Trizol (Invitrogen) according to the manufacturer’s instructions.

To quantify mRNA, real-time PCR was performed as previously reported^62^. In brief, a total of 1 μg RNA was reverse-transcribed using a cDNA synthesis kit (Invitrogen) according to the manufacturer’s instructions. For real-time PCR, 2 μl template cDNA was mixed with 5 μl SYBR Green Supermix, 0.4 μl forward primer, 0.4 μl reverse primer, and 2.2 μl ddH_2_O. The cycling conditions were held as follows: 95 °C for 10 min, followed by 40 cycles at 95 °C for 15 s and 60 °C for 1 min. The fluorescent signals were analyzed using an ICycler IQ5 detection system (Bio-Rad). The relative level of uc.372 was determined using the 2^−delta delta Ct^ analysis method. β-actin was applied as the endogenous control. The primers used for real-time PCR are listed in Supplementary Table 1.

### RNA-fluorescence in situ hybridization

The probes tiling the uc.372 RNA and U6 were designed following Stellaris^®^ RNA-fluorescence in situ hybridization probe designer (Biosearch Technologies). Cell fixation, permeabilization, and hybridization to probes were performed following protocols for adherent cells. Images were acquired on a Zeiss Axio Observer Z1 Apotome fluorescence microscope.

### RNA-RIP assay

RIP assay was carried out to explore the interaction between Drosha, pri-miR-195 or pri-miR-4668, and uc.372 as previously described^63^. Briefly, cells were seeded at a density of 1 × 10^5^ cells/ml for 24 h. Then, the cells were transfected with Ad-NC or Ad-uc.372m for 48 h. After that the cells were incubated with glycine after fixing by formaldehyde (0.3%). The cells were washed with phosphate-buffered saline (PBS) for three times and cell suspension was reserved in RIP buffer. Then, anti-Drosha antibody (Abcam, USA) was added and incubated with the cell suspension at 37 °C overnight. TRIzol reagent (Invitrogen) was used to isolate the precipitated RNA and the level of pri-miR-195 or pri-miR-4668 was further analyzed using real-time PCR.

### Luciferase reporter assay

The 3′UTR of SCD1 and CD36 containing the predicted binding site for miR-4668 was cloned into the pmirGLO (Promega, Madison, Wisconsin, USA) luciferase reporter vector. PCR procedures were performed as previously described. Transfection was carried out using Vigofect transfection Reagent (Vigorous Biotechnology, Beijing, China) according to the manufacturer’s recommendations. Luciferase reporter assays were performed using the Dual Luciferase Reporter Assay System (Promega). Renilla activity was used as the internal control.

### Northern blot

A unit of 30 μg of RNA was mixed with denaturing RNA load dye (1:1, v:v, Solarbio, Beijing, China) and heated to 95 °C for 5 min. The RNA samples was separated on 8% denaturing Trisborate-EDTA-Urea (TBE-Urea) polyacrylamide gels and then transferred onto positively charged nylon membranes (EMD Millipore, Billerica, MA, USA). A Spectrolinker XL-1000 UV crosslinker (Xinzhi, Ningbo, China) was used for the crosslinking of RNA on the membrane at 240 mJ/cm^2^. Then, the membranes were incubated with double-digoxigenin LNA probes in Ultrahyb Oligo hybridization buffer (Mylab Medical Technology, Beijing, China) with 2× blocking reagent (Mylab Medical Technology) at 60 °C overnight. After washing with 2×, 0.5×, and 0.1× saline sodium citrate buffer containing 0.1% SDS for 20 min at 60 °C, the membranes were blocked with 100 mM maleic acid, 150 mM NaCl, pH 7.5, with 2× blocking reagent for 30 min. After that the membranes were incubated with primary antibody (Mouse anti-Dig, Mylab Medical Technology) followed by three washes of PBS containing 0.1% Tween 20 (PBS-T). The blot was further incubated with secondary antibodies (goat anti-mouse IGG, Mylab Medical Technology) followed by detection with chemiluminescence (Xinzhi). Probe sequences were as follows:

uc.372-5′ probe, CCTTCCTATACAGCCATCCCC;

uc.372-3′ probe, CTTAGAACAATCTGCCATTTGGA.

### Western blot analysis

Cells or liver tissues were extracted with RIPA buffer (Solarbio). Approximately 15–30 µg of proteins were separated by 10% SDS-PAGE and further transferred onto a polyvinylidene difluoride membrane (Millipore, Boston, MA, USA). The membrane was soaked with 8% milk in PBS-T (pH 7.5) for 2 h at room temperature and was incubated with the following specific primary antibodies at 4 °C overnight: anti-ACC (1:1000, #3676, CST); anti-FAS (1:1000, #3180, CST); anti-SCD (1:1000, #2283, CST); anti-CD36 (1:1000, ab133625, Abcam); and anti-GAPDH (1:1000, #5174, CST). After washing three times with PBS-T, the membrane was incubated with the horseradish peroxidase-conjugated anti-rabbit or anti-mouse secondary antibody (1:5000, Zhongshan Gold Bridge, China) for 2 h at room temperature. Immunodetection was conducted using the ECL Plus detection system (Millipore) according to the manufacturer’s instructions. The housekeeping gene *GAPDH* was used as the internal control.

### Triglyceride measurement

A unit of 20 mg of liver tissues was moved to pre-weighed glass tube. Then, 4 ml CHCL_3_/CH_3_OH (2:1) was added and incubated overnight at room temperature. By morning, 850 μl 0.05% H_2_SO_4_ was added and votexed. After centrifugation for 15 min at 2700 rpm at room temperature, we moved supernatant and added 1% Triton X-100. The mixture was put in vacuum evaporator at 60 °C overnight. After the mixture was completely dry, 1 ml ddH_2_O was added to each tube at 55 °C. Then, we used a triglyceride enzymatic assay kit (ShenSuoYouFu Medical Diagnostic Products Co., Ltd., Shanghai, China) to determine the contents of triglyceride.

### Oil Red O staining

Frozen sections of liver specimens were fixed in paraformaldehyde. Then, the slides were stained with Oil Red O as previously described^64,65^.

### Hematoxylin and eosin staining

For hematoxylin and eosin staining, the slides were first incubated with hematoxylin for 5 min and then washed with 1% ethanol hydrochloride for 3 s. After washing with water, the slides were stained with eosin for 3 min and dehydrated with an alcohol gradient. Vacuoles were considered the lipid droplets^65^.

### Cell fractioning

Cytoplasmic and nuclear fractions were obtained with the ProteoExtract Subcellular Proteome Extraction Kit (Calbiochem, #539790). RNA from each fraction was extracted with TRIzol.

### Statistical analysis

The data are represented as the mean ± standard error of the mean. Comparisons between groups were analyzed by the two-tailed Student’s *t*-test. For comparisons of multiple groups, one-way analysis of variance was used. Differences were considered significant at *P* < 0.05.

### Data availability

The microarray data discussed in this publication have been deposited in NCBI’s Gene Expression Omnibus (GEO) and are accessible through GEO Series accession number GSE106366, GSE107231, and GSE107396. All data pertinent to this study are available in Supplementary Information. Additional information on these data is available from the corresponding authors.

## Electronic supplementary material

Supplementary Information

Description of Additional Supplementary Files

Supplementary Data 1

Supplementary Data 2

Supplementary Data 3

Supplementary Data 4

Supplementary Data 5

Supplementary Data 6

Supplementary Data 7

Supplementary Data 8
