# Supplementary material for: Ultraconserved element uc.372 drives hepatic lipid accumulation by suppressing miR-195/miR4668 maturation
Source: Nat Commun. 2018 Feb 9;9:612. doi: 10.1038/s41467-018-03072-8 (PMC5807361; doi:10.1038/s41467-018-03072-8)
Supplement: Supplementary file 1 — Supplementary Information [file 41467_2018_3072_MOESM1_ESM.pdf]

Supplementary Table 1： A list of real-time PCR primers used

Gene Name	Forward Primer	Reverse Primer
usf1 (Mouse)	TGCGGGCCTAGTACTTCCA	CCCCTGCCTACAGTTCTCC
usf2 (Mouse)	TTGCCTCAATCTAAGTACTCC	TTATATGAGTACACGGTCGAT
evi-1 (Mouse)	TTGGCTTTTCTTAACATTCCT	TGAACATAACATCATCGCACA
elk-1 (Mouse)	CAAACGGTCACCTTTTACCAC	TGGCTCACACAATCAGCTT
p54 (Mouse)	ATCTCTATCAGGAATACTGCC	CATTTAAGACACTTACATCGCTA
chop-c (Mouse)	CCCCACTCAGCTTACAACA	ATCGATTGTGCTTCAAGTTCC
cp2 (Mouse)	TTGTAAAGCACTTAGGCCCTC	AAAACTTTACATGTGGCCTGAC
oct-1 (Mouse)	TTTAATTACTATTGATTCAGGGG	GACACCCAATAAACTACCC
fas (Mouse)	GTTGGCCCAGAACTCCTGTA	GTCGTCTGCCTCCAGAGC
acc (Mouse)	GGAGGACCGCATTTATCGA	TGACCAGATCAGAGTGCCT
scd1 (Mouse)	CTACTTCAAGGGCAGTTCTGA	AAGGTTTCCTGCAATGGTTT
cd36 (Mouse)	ATGGGCTGTGATCGGAACTG	GTCTTCCCAATAAGCATGTCTCC
β-actin (Mouse)	GGCTGTATTCCCCTCCATCG	CCAGTTGGTAACAATGCCATGT
insm2 (Mouse)	GCAACAAGATGTAAAACCCC	TTTTATGCCAAATACATACCTG
actb (Mouse)	AACATCCCCCAAAGTTCTAC	GGACTTCCTGTAACCACTTA
nup62 (Mouse)	TTGTGCCTAAGTGGTAAGTC	GAGCGACTACCTCTCTATCT
ralgapa1 (Mouse)	ATGTATCTACACGAATGCCTT	GTTCTGACCAAACAATGTGC
β-actin (Mouse)	TGGTTACAGGAAGTCCCTC	AGCCTTCATACATCAAGTTGG
FAS (Human)	CTTGGTCTTCTTTATTGGCAT	AGGAAAATTACAAATGGCCTT
ACC (Human)	TTGATTCCTGGCTCTACCC	TCACTGCCTCTGAATACACA
SCD1 (Human)	TTGATTCCTGGCTCTACCC	TCACTGCCTCTGAATACACA
SREBP1 (Human)	TCTCTTAGAGCGAGCACTGA	TCAGAGAGGCCCACCACTT
LXR (Human)	CTATCGGCTCTCATCCCTT	GACCTGCAACCCTTTCACC
Fatp1 (Human)	GATGTCCCCATTTAGCCAT	TATTCAACAGGCTAGAACCCC
Fatp2 (Human)	CCGGTTTCTAAGAATACAGG	ATCCAAGAAATACAAGGCAT
Fatp5 (Human)	CCCATTTCATCCGCATCCAG	TGGTACATTTCTGCCGTCA
CD36 (Human)	AACCTATTGGTCAAGCCAT	ATGTTTGCCTTCTCATCACC
PPAR-γ(Human)	AGCCTCATGAAGAGCCTTCCAACT	TGTCTTTCCTGTCAAGATCGCCCT
Fabp1 (Human)	AACATCAAGTCTGTGACCGAA	TTGAAGACAATGTCACCCAA
Cpt1α(Human)	TAGATCATGCACTGTTGACCA	TATGTTTAAGCACTCACGTCT
Scad (Human)	TCAAGTTCCTCATCTAAGTGGC	AAGGAAAAGACAGACCCCA
Acox1 (Human)	CGGACTACACTTCATAAATGCC	GCAAAGTAATTATGTGCTCCC
PPAR-α(Human)	AGTCTCCCAGTGGAGCATTGAACA	ATACGCTACCAGCATCCCGTCTTT
apoB (Human)	ATATCTTAGCATCCTTACCGA	AAATTATTTTCTTCGTCGCAAT
Mtp (Human)	AAAATATTGGACCTAGCACAG	GTCATCCTAGCTATTGTGCAG
β-actin (Human)	CGGGTCACCCACACTGTGC	CTAGAAGCATTTGCGGTGGACGATG
pri-miR-195 (Human)	AGCTTCCCTGGCTCTAGCA	ATATTGGCAGACTCGCTTCCC
miR-195 (Human)	TAGCAGCACAGAAATAT	GTGCAGGGTCCGAGGT
pri-miR-4668 (Human)	AAAAAGGATTTGTCTTGTAG	ATTTTCTTTAAAACAATATCCTG
miR-4668 (Human)	GCAGGGAAAAAAAAAAGGATT	GTGCAGGGTCCGAGGT
U6	GCGCTCGTGAAGCGTTC	GTGCAGGGTCCGAGGT

ACTB (Human)	TCCAAATATGAGATGCGTTG	CCTTAGAGAGAAGTGGGGT
Nup62 (Human)	ACGTCAGTCCACAGAAATAG	AGTTACTCTGGTGCCACTAA
RALGAPA1 (Human)	ACGCATTCTTAACATTTATCGG	ATCCAGGCAACTATAAGGGTC
uc.348	AAGCCTTTTAAATGCAGTCT	TCTATGGCACTGTAAACTCA
uc.372	AGCCAGATCTAATACTAAGCTC	ATCACTTAGAACAATCTGCC
uc.393	ATCACTTAGAACAATCTGCC	GGCACACAGCATATATAACGA
uc.294	CTCATCCGAGTGTGTTAAGCA	TTAAGAAATTCGCTCGCCAT
uc.53	CTCATTAAATTAGGCCACA	TCTTCATCCATTTAGCCAT
uc.94	TAAATGATGTATTTAGAGCAGT	ACCAACACACTAGGGGAC
uc.361	GCACAATTTGTACAGACC	CATGCTCATAAGTGCAAG
uc.140	ATAAATTGTAATACCGTTGC	AGTTTATTATACAAGGCTCT
uc.343	TCCCTTTTATTCGCCACA	GAACTAAAATCCTGAGCGAT
uc.157	GATTAAACCGTGATTTAGCC	TCATTAAGGAAATCGCTCT
uc.436	GGCACACAGCATATATAACGA	GATTTTCTGATTGCGGTT
uc.349	CTAATTTAGTTGCACTCCT	GTTGATTAGAATTCAAGCCT
uc.351	AATACATTATGTGCATACAAA	CTTCTTAACATTGTCCCAT
uc.72	ATAAAACTCGCTTAGCAG	AAGAAATTTAAGCACGGAA
uc.227	ATTTTCAGCCTATAATCCAA	ATCAAACGCAATTAGCAA
uc.402	CCACTAATACAGGTACAAGGC	AGTTGAAACCGAGTAACCTTT
miR-4668-RT	GTCGTATCCAGTGCAGGGTCCGAGGTATTCGCACTGGATACGAC GACAAAT	
miR-195-RT	GTCGTATCCAGTGCAGGGTCCGAGGTATTCGCACTGGATACGAC GCCAAT	
U6-RT	GTCGTATCCAGTGCAGGGTCCGAGGTATTCGCACTGGATACGAC AAATATG	

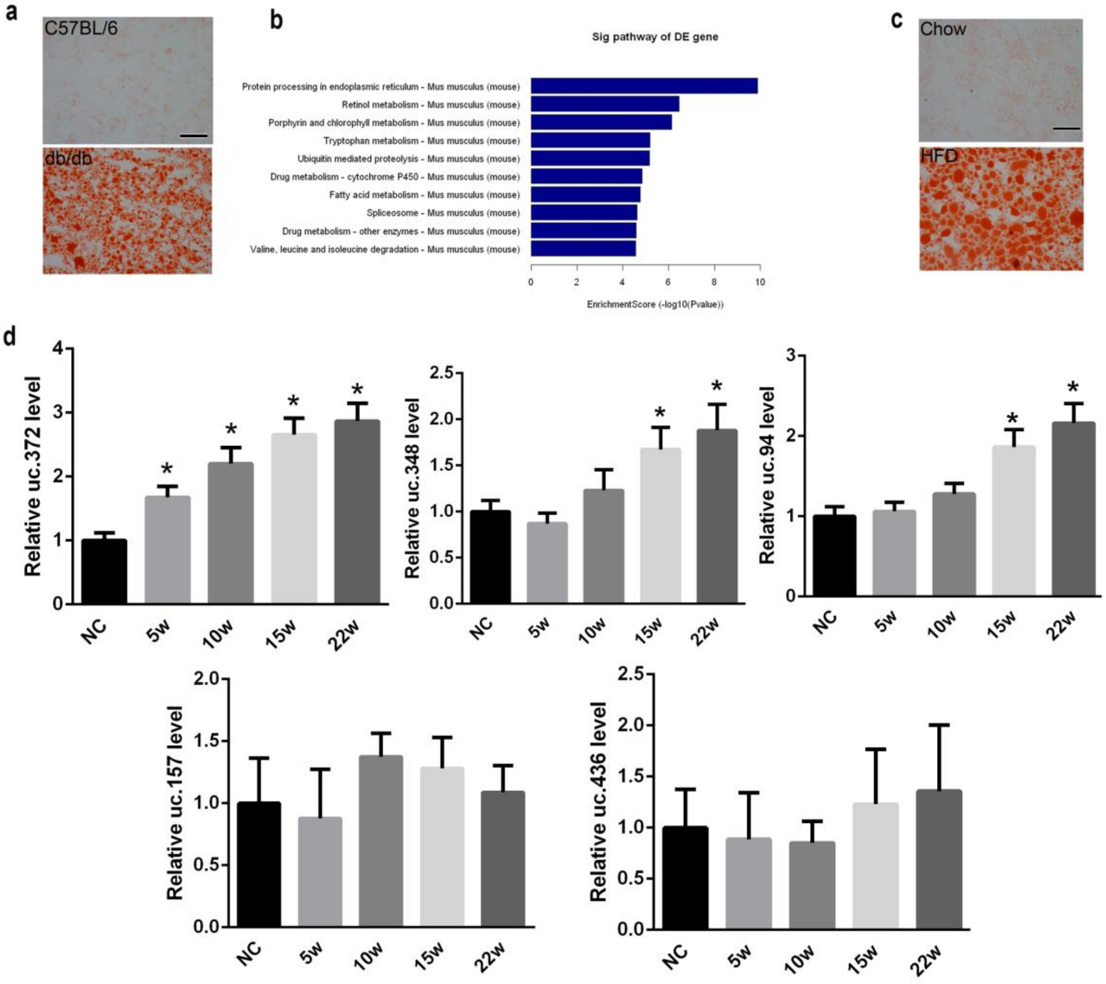

Supplementary Figure 1, related to Figure 1. *uc.372 correlates with abnormal hepatic lipid* accumulation. **a** Representative image from 3 similar experiments of Oil Red O staining in the liver of eight-week-old male db/db mice. Scale bar, 100μm. **b** KEEG pathway. **c** Representative image from 3 similar experiments of Oil Red O staining in the liver of ten-week-HFD-fed mice. **d** The expression of uc.348, 372, 94, 157 and 436 in five-, ten, fifteen-, and twenty two-HFD-fed mice (n=5). Data are mean ± SEM; *P< 0.05 vs. control group. ((d), analysis of variance (ANOVA)).

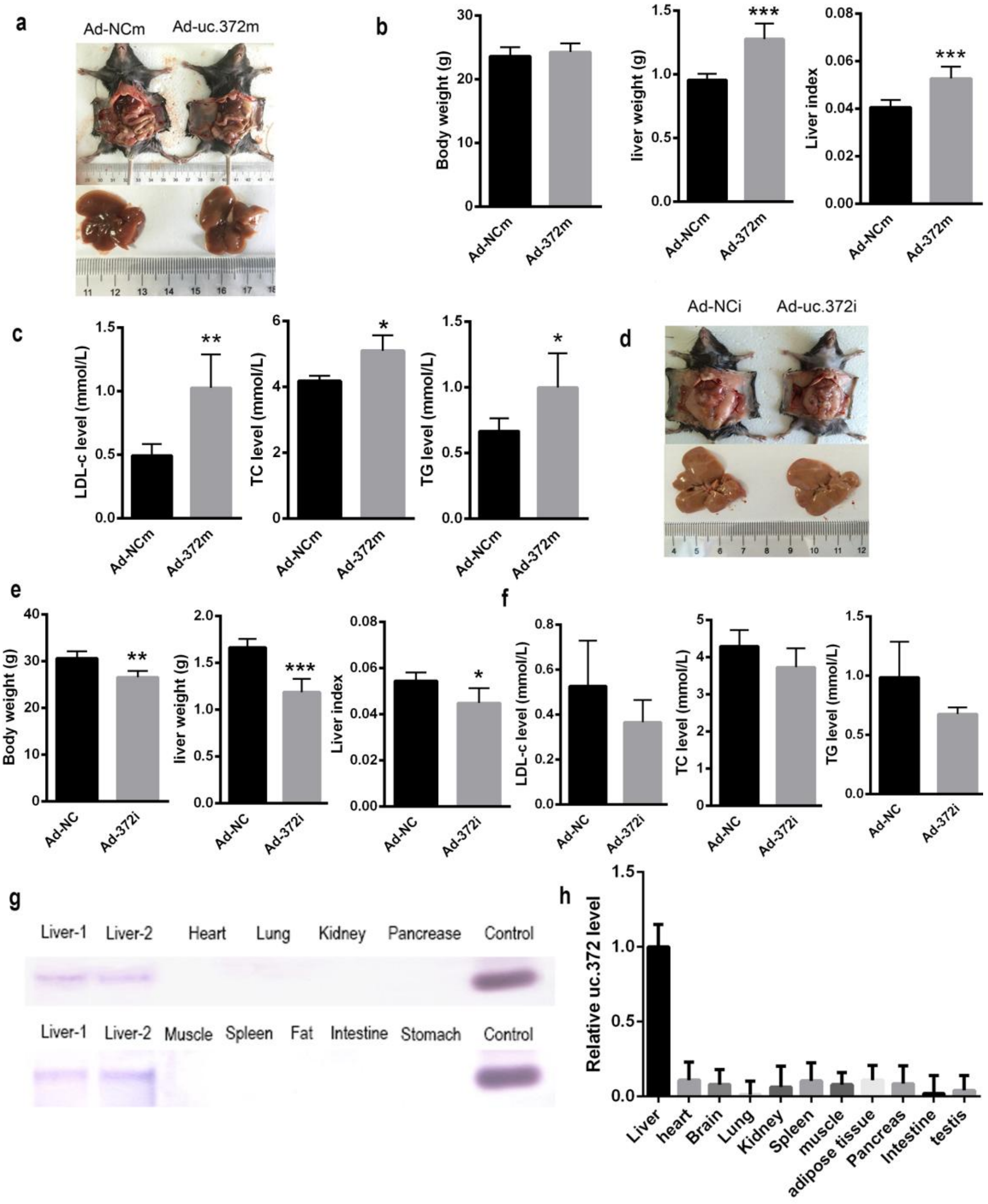

Supplementary Figure 2, related to Figure 2. *uc.372 plays critical role in hepatic lipid metabolism*. **a** The liver of six-eight-week-old C57BL/6J mice injected with Ad-uc.372m or Ad-NCm for 7 days. **b** Body weight, liver weight and liver weight-to-body weight ratio in C57BL/6J mice injected with Ad-uc.372m (n=5). **c** Plasma LDL-c, TG and TC levels in six-eight-week-old C57BL/6J mice injected with Ad-uc.372m or Ad-NCm for 7 days (n=5). **d** The liver of ten-week-HFD-fed mice injected with Ad-uc.372i or Ad-NC for 7 days. **e** Body weight, liver weight and liver weight-to-body weight ratio in ten-week-HFD-fed mice injected with Ad-uc.372i or Ad-NC for 7 days (n=5). **f** Plasma LDL-c, TG and TC levels in ten-week-HFD-fed mice injected with Ad-uc.372i or Ad-NC for 7 days (n=5). **g** The

expression of uc.372 in different tissues after tail vein injection with Ad-uc.372m was analyzed by Northern blot. PCR products of uc.372 were used as the positive control. **h** The level of uc.372 in different tissues after seven days injection of ad-uc.372m (n=3). Data are mean ± SEM; ((b, c, e, f) Student's t test; (h), analysis of variance (ANOVA)): *P< 0.05; **P< 0.01; ***P< 0.001 vs. control group.

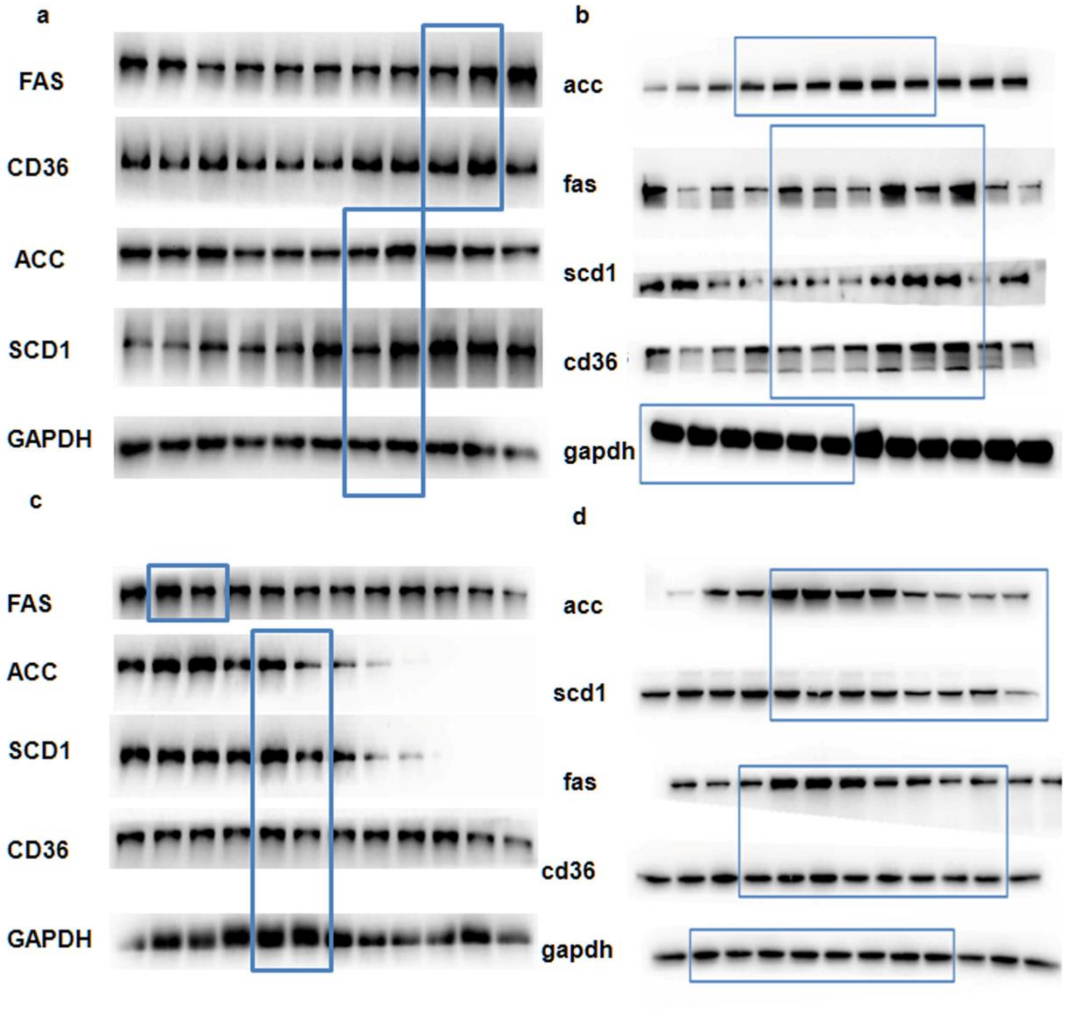

Supplementary Figure 3, related to Figure 3. The *uncropped scans of the western blots related to Fig. 3b, 3d and Fig. 3f, 3h.*

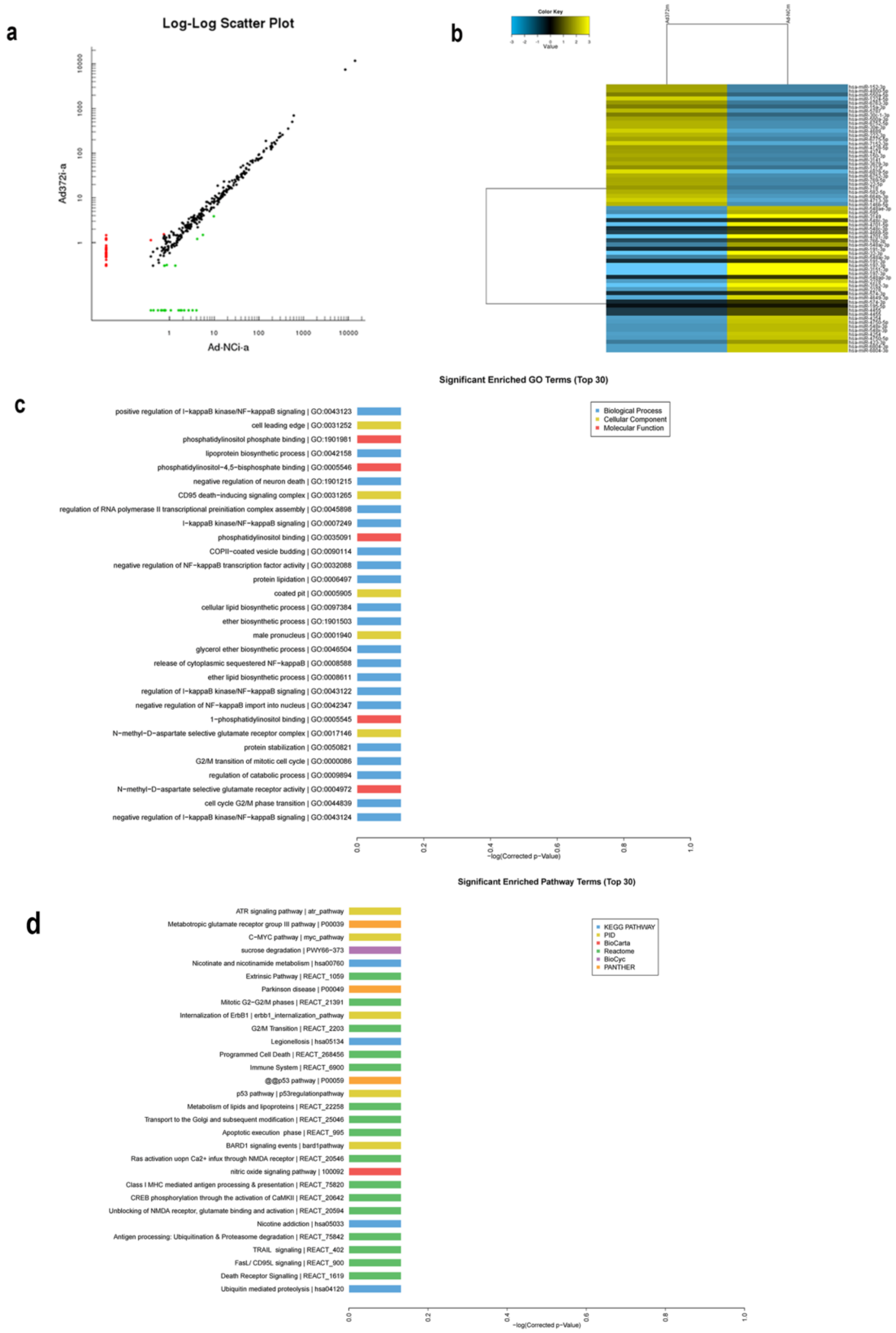

Supplementary Figure 4, related to Figure 4. *Identification of miRNAs regulated by uc.372.* **a** Scatter plot analysis. **b** Microarray analysis. **c** GO analysis. **d** KEEG pathway analysis.

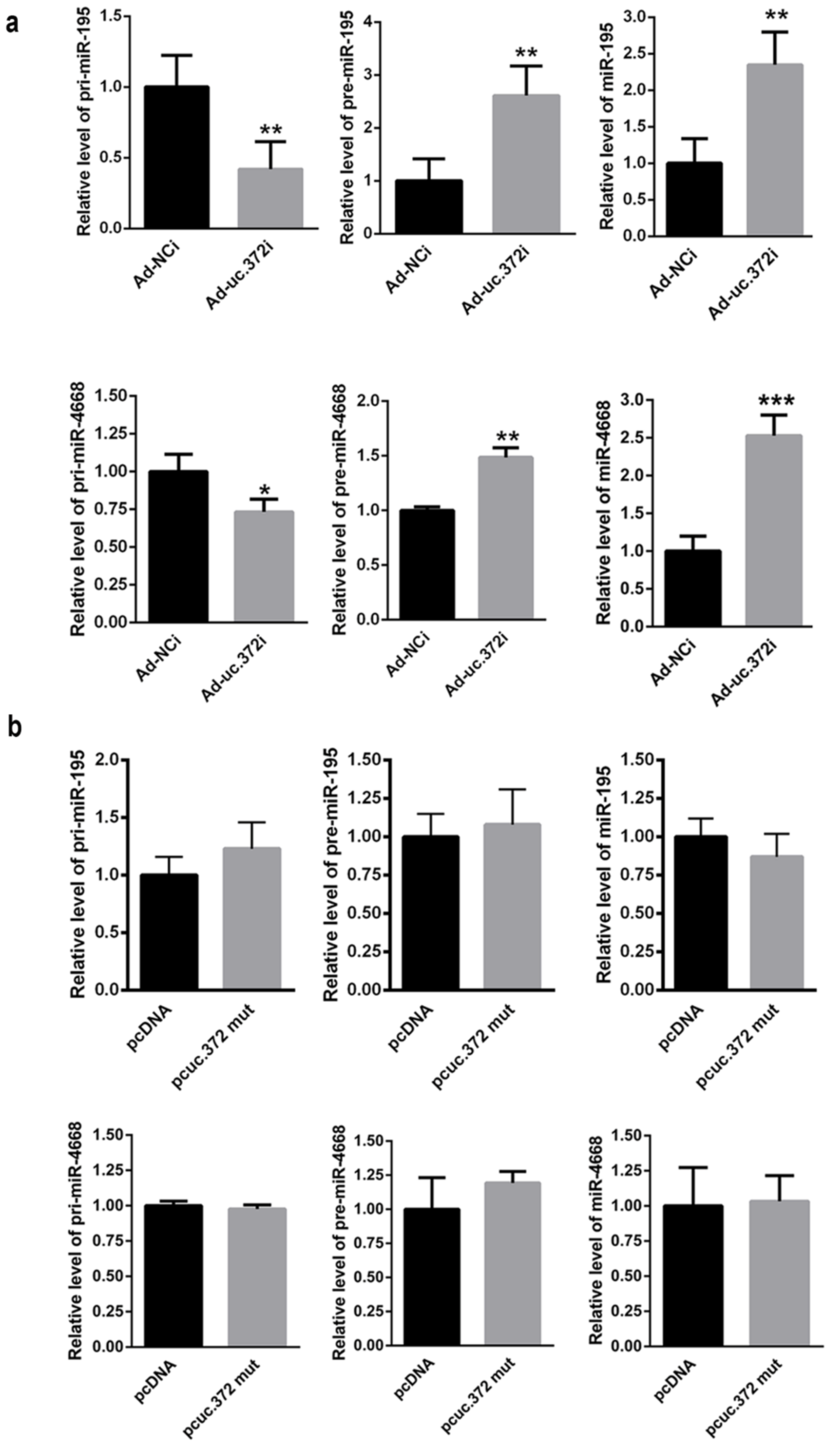

Supplementary Figure 5, related to Figure 4. *Uc.372 Binds to Pri-miR-195/Pri-miR-4668.*

a The levels of pri-miR-195/pri-miR-4668, pre-miR-195/pre-miR-4668 and mature miR-195/miR-4668 in the HepG2 cells transfected with Ad-uc.372i or Ad-NCi for 48 h (n=3).

b The levels of pri-miR-195/pri-miR-4668, pre-miR-195/pre-miR-4668 and mature miR-195/miR-4668 in the HepG2 cells transfected with pcuc.372 mutant or pcDNA for 48 h (n=3). Data are mean ± SEM; (a, b) Student's t test: *P< 0.05; ***P< 0.001 vs. control group.

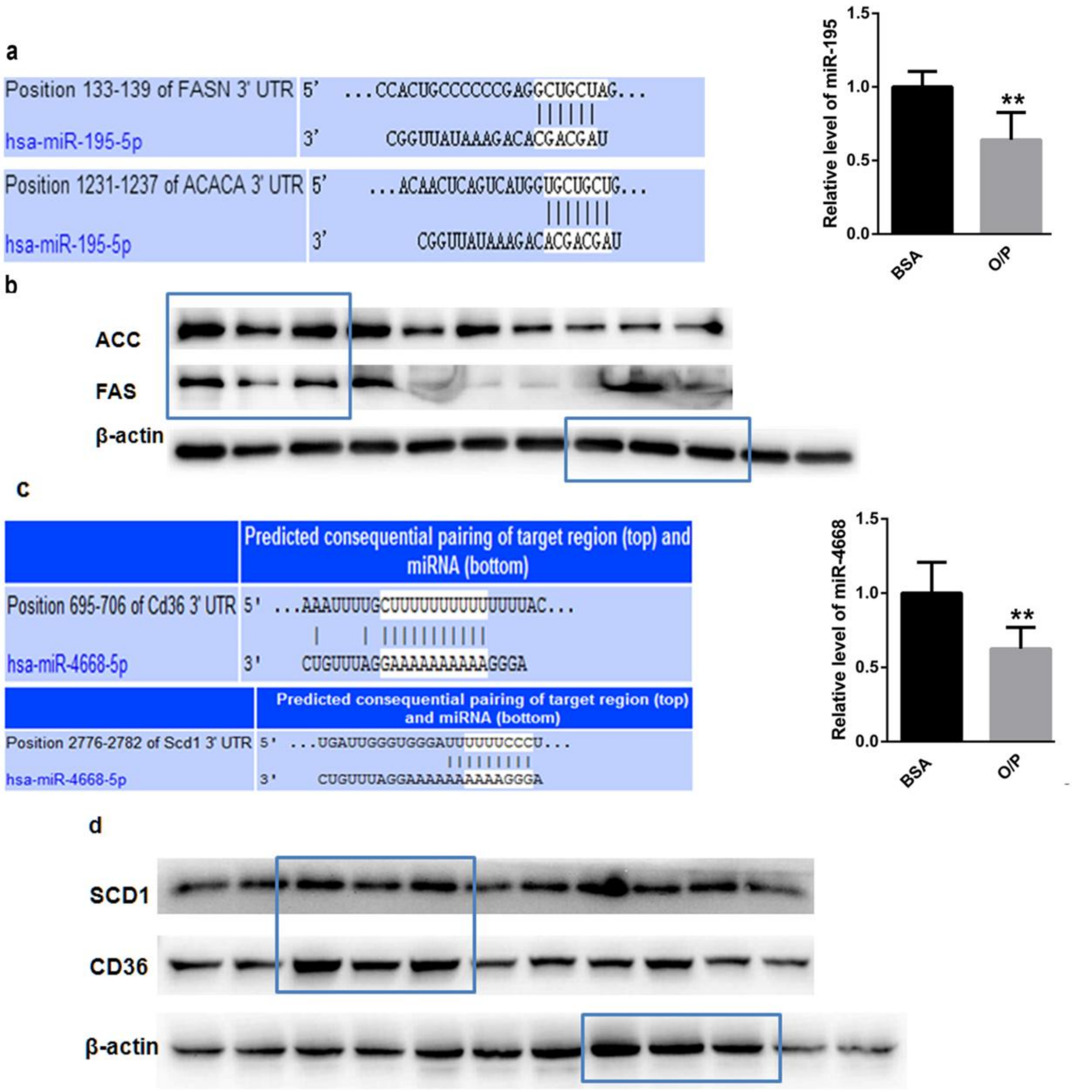

Supplementary Figure 6, related to Figure 5. *ACC and FAS or SCD1 and CD36 are targets of miR-195 or and miR-4668.* **a** (Left panel) The binding sites of miR-195 in the 3'UTR of ACC and FAS. (right panel) The level of miR-195 in HepG2 cells treated with 300 μM O/P mixture for 48 h (n=3). **b** The uncropped scans of the western blots related to Fig. 5c. **c** (Left panel) The binding sites of miR-4668 in the 3'UTR of SCD1 and CD36. (right panel) The level of miR-4668 in HepG2 cells treated with 300 μM O/P mixture for 48 h (n=3). **d** The uncropped scans of the western blots related to Fig. 5g. Data are mean ± SEM; (a, c) Student's t test:*P< 0.05; **P< 0.01 vs. control group.

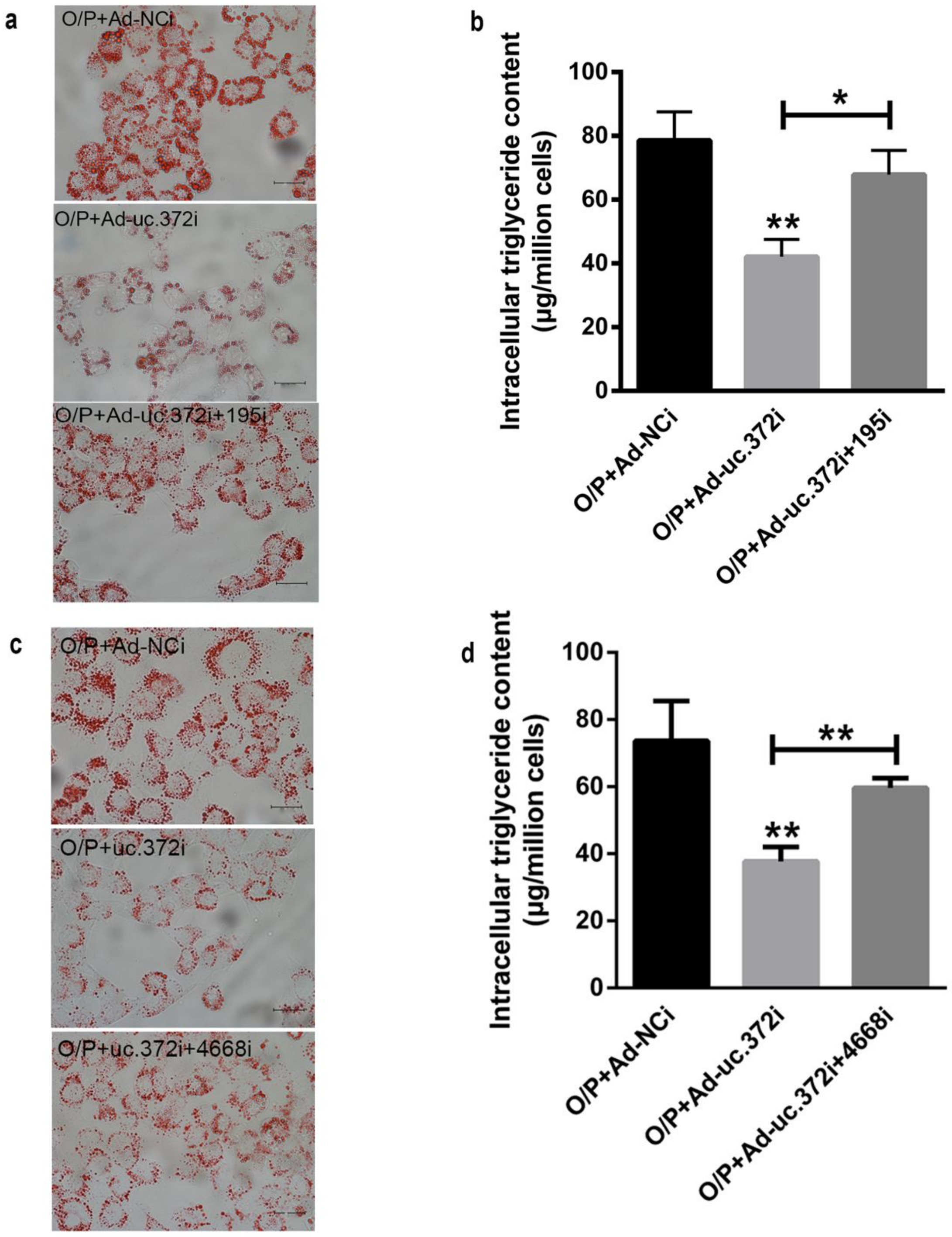

Supplementary Figure 7, related to Figure 5.

a Representative image from 3 similar experiments of Oil Red O staining in Ad-uc.372i infected-and miR-195 inhibitor-transfected HepG2 cells pre-treated with 300 μM O/P mixture for 48 h. Scale bar, 25μm.

b Intracellular triglyceride content in Ad-uc.372i-infected and miR-195 inhibitor-transfected HepG2 cells pre-treated with 300 μM O/P mixture for 48 h (n=3).

c Representative image from 3 similar experiments of Oil Red O staining in Ad-uc.372i infected-and miR-4668 inhibitor-transfected HepG2 cells pre-treated with 300 μM O/P mixture for 48 h. Scale bar, 25μm.

d Intracellular triglyceride content in Ad-uc.372i-infected and miR-4668 inhibitor-transfected HepG2 cells pre-treated with 300 μM O/P mixture for 48 h (n=3). Data are mean ± SEM; *P< 0.05; **P< 0.01 vs. control group. ((b, d) analysis of variance (ANOVA)).

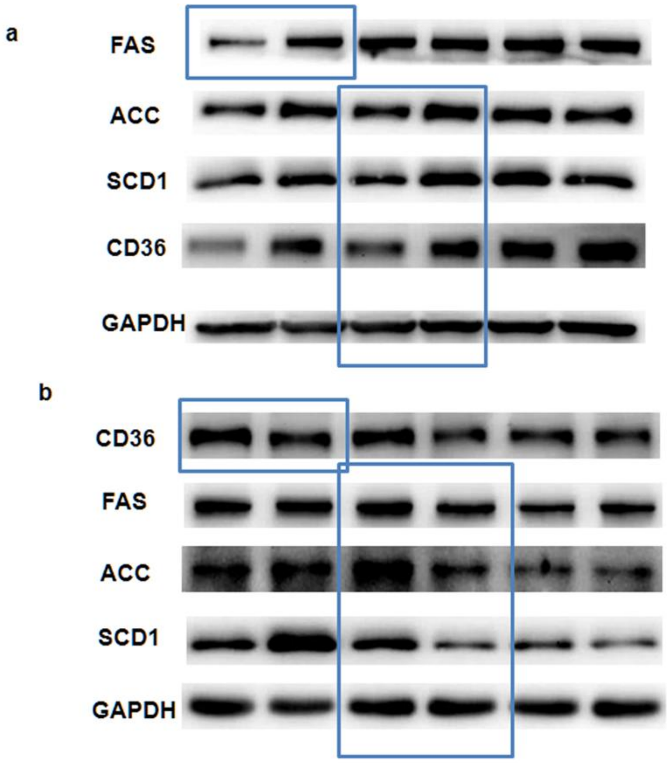

Supplementary Figure 8, related to Figure 7.

The uncropped scans of the western blots related to Fig. 7e and 7g.

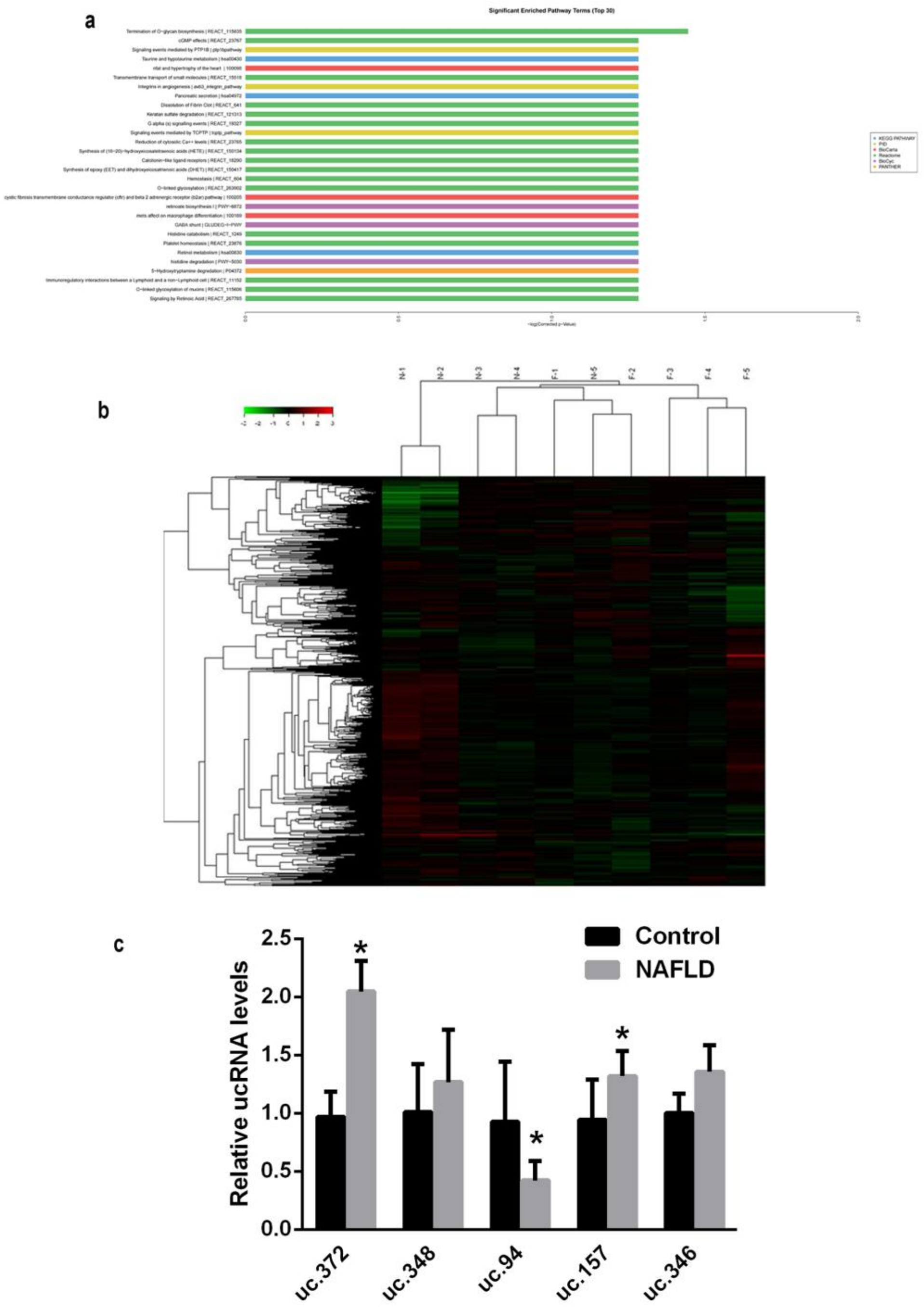

Supplementary Figure 9, related to Figure 8. *Identification of uc.372 expression in the liver of NAFLD patients*. **a** KEEG pathway analysis. **b** LncRNA-wide expression profiling. **c** The expression of uc.348, 372, 94, 157 and 436 in the liver of NAFLD patients and healthy control (n=5). Data are mean ± SEM; ((c) Student's t test): *P< 0.05 vs. control group.
