# Supplementary material for: Ultraconserved element uc.372 drives hepatic lipid accumulation by suppressing miR-195/miR4668 maturation
Source: Nat Commun. 2018 Feb 9;9:612. doi: 10.1038/s41467-018-03072-8 (PMC5807361; doi:10.1038/s41467-018-03072-8)
Supplement: Supplementary file 2 — Description of Additional Supplementary Files [file 41467_2018_3072_MOESM2_ESM.pdf]

Description of Additional Supplementary Files

File Name: Supplementary Data 1
Description: Raw data related to fig. 1b, 1c, 1d and 1e. The raw data included raw Ct value for each sample. The calculation was carried out using the 2-delta delta Ct analysis method.

File Name: Supplementary Data 2
Description: Raw data related to fig. 2a, 2c, 2g, 2i. The raw data included raw Ct value for each sample in fig. 2a and fig. 2g. And the calculation was carried out using the 2-delta delta Ct analysis method. For fig. 2c and 2i, the raw data were the hepatic glyceride content for each sample

File Name: Supplementary Data 3
Description: Raw data related to fig. 6b, 6c. The raw data included raw Ct value for each sample. The calculation was carried out using the 2-delta delta Ct analysis method.

File Name: Supplementary Data 4
Description: Raw data related to Fig. 7a, 7b. The raw data included raw Ct value for each sample. The calculation was carried out using the 2-delta delta Ct analysis method.

File Name: Supplementary Data 5
Description: Raw data related to fig. 8d, 8e. The raw data included raw Ct value for each sample. The calculation was carried out using the 2-delta delta Ct analysis method.

File Name: Supplementary Data 6
Description: Raw data related to Fig. S1d. The raw data included raw Ct value for each sample. The calculation was carried out using the 2-delta delta Ct analysis method.

File Name: Supplementary Data 7
Description: Raw data related to Fig. S2. Raw data of liver weight, body weight, and liver/body index (b, e). Raw data of TG, TC, LDL-c (c, f).

File Name: Supplementary Data 8
Description: Raw data related to Fig. S9C. The raw data included raw Ct value for each sample. The calculation was carried out using the 2-delta delta Ct analysis method.
